# Supplementary material for: Rapid electron transfer via dynamic coordinative interaction boosts quantum efficiency for photocatalytic CO2 reduction
Source: Nat Commun. 2021 Jul 13;12:4276. doi: 10.1038/s41467-021-24647-y (PMC8277789; doi:10.1038/s41467-021-24647-y)
Supplement: Supplementary file 1 — Supplementary Information [file 41467_2021_24647_MOESM1_ESM.docx]

Supplementary Information

Jia-Wei Wang et al.

**Experimental details**

**Synthesis of IrQPY**

After obtaining the chloride salt, [Ir(qpy)(ppy)_2_]Cl,^1^ it was dissolved in water as a saturated solution. Excess HBF_4_ was dropped in the above solution until no more precipitation took place. After filtration, the solid was dried under vacuum and darkness, affording the orange powder of IrQPY as the product (94% yield). The orange block crystals were isolated by evaporation of a chloroform solution of IrQPY. ESI-MS(+) from Q-TOF: [Ir(qpy)(ppy)_2_]^+^ (measured: 811.2161; simulated: 811.2156). ^1^H NMR (2.0 mM, 400 MHz, DMF-*d*_7_): δ 9.67 (d, *J* = 1.5 Hz, 2H), 8.86 (dd, *J* = 4.5, 1.6 Hz, 4H), 8.46 – 8.38 (m, 2H), 8.28 (dd, *J* = 5.8, 1.8 Hz, 2H), 8.21 (d, *J* = 5.8 Hz, 2H), 8.12 (dd, *J* = 4.5, 1.7 Hz, 4H), 8.07 (m, *J* = 3.5, 2.1 Hz, 6H), 7.31 – 7.21 (m, 2H), 7.15 – 7.08 (m, 2H), 7.00 (td, *J* = 7.4, 1.2 Hz, 2H), 6.39 (d, *J* = 6.8 Hz, 2H). Elemental analysis: Calculated (IrC_42_N_6_H_30_BF_4_), C, 56.19; H, 3.37; N, 9.36; Measured, C, 56.26; H, 3.41; N, 9.44.

**NMR titrations and determination of binding constant**

A 0.5 mL solution of host (2.0 × 10^−4^ or 1.0 × 10^−3^ M) in DMF-*d*_7_ was titrated with another solution containing the same concentration of host (2.0 × 10^−4^ or 1.0 × 10^−3^ M) and more concentrated guest (1.0 × 10^−3^ M or 2.5 × 10^−2^ M) in DMF-*d*_7_. Upon each addition, the solution was manually stirred for 3 min before acquiring the spectrum, which allowed equilibrium to be reached between the host and guest.

Binding isotherms for the NMR titration were calculated from a global proton shift analysis (using proton signals from the qpy ligand in IrQPY) using BINDFIT with the shift differences (*δ* - *δ*_0_) taken to obtain a binding isotherm.^2^ The fitting is based on the shift difference between the host-only data and the data at varying guest concentrations. The equations used for these analyses are available in the review by Thordarson.^3^ Nelder-Mead (Simplex) method was used as the optimal search algorithm. The error of binding constant, the root mean square and the covariance of the fit (variance of the residuals divided by the variance in the data) were used to conclude the models that best describe the binding between IrQPY and metal complexes. Specific discussion on the choices of the models is included in the table captions. It should be noted that the non-cooperative mode means that each of the multiple binding process does not interfere with each other, in which the second binding constant (*K*_12_ or *K*_21_) will be equivalent to the first binding constant (*K*_11_).

**DFT calculation**

All the calculations were performed with Gaussian 09 program.^4^ All the structures were fully optimized at the M06^5^/BSI level of theory in gas phase (BSI designated the basis set combination of Lanl2DZ^6^ for Co and Ir atoms, 6-31G(d) for nonmetal atoms). Frequency analysis calculations were performed to characterize the structures to be the minima. With M06/BSI optimized geometries, the energy results were refined by single-point calculations at the M06/BSI level of theory. The Co(II) center is calculated as low-spin for its lower energy by 20.6 kcal mol^-1^ than that of its high-spin state, consistent with the previous reports.^7^ The Ir(III) atom should also be low-spin for the sharp peaks in its ^1^H NMR spectra. The solvation effect of acetonitrile was also evaluated with the SMD solvent model at the BP86/BSII (BSII designated the basis set combination of Lanl2DZ for Co and Ir atoms, 6-311+G(d) for nonmetal atoms) level of theory. A concentration correction of 1.9 kcal mol^-1^ was added to the free energy values for all the species to obtain the results at the standard state of 1.0 M solution since the frequency calculations were carried out with the ideal gas model (1 atm, 24.5 M, 298.15 K). The 3D optimized structure figures in this paper were drawn by Olex2 program. The Mayer bond order analysis is performed with Multiwfn^8^ program.

**Cyclic voltammetry**

Cyclic voltammetry was performed in a 25 mL gas-tight three-neck flask with a three-electrode system, where a 3 mm glassy carbon disc electrode, a Pt wire counter electrode, and a Pt wire pseudo-reference electrode were placed in a 0.1 M tetrabutylammonium hexafluorophosphate CH_3_CN solution (8.0 mL). In the cases of CoPc and CoTAPc, *N*-methyl pyrrolidone/CH_3_CN mixed solvent (v:v = 1:1; 8.0 mL) was used for a better solubility. The solution was bubbled with N_2_/CO_2_ for 15 min prior to each experiment. Scan rate is 100 mV s^-1^. All potentials were referenced against ferrocenium/ferrocene (Fc^+^/Fc) as an internal standard. Unless otherwise stated, all potentials were footnoted as vs. Fc^+^/Fc.

**Photocatalytic experiments**

The photocatalytic reduction of CO_2_ to CO was conducted in a 17 mL home-made reactor upon successive addition of catalyst, proton source, a CH_3_CN solution containing Ir PS and BIH, and finally TEA, under 1 atm CO_2_ atmosphere at 293±2 K. PhOH was placed in a N_2_-saturated Schlenk flask and heated to liquid state at 70 °C prior to use. It should be noted that CoPc and CoTAPc are not fully soluble in a concentrated mixture (5 mM) with the use of NMP, but it became homogeneous when the above mixture was sonicated and diluted in the photocatalytic system. After the reaction system purged with CO_2_ for 10 min, the photocatalytic reaction was initiated by irradiation under an LED light (typically λ = 450 ± 5 nm, light intensity = 100 mW cm^-2^, irradiated area is 0.80 cm^2^). The generated gases in the headspace were analyzed by a gas chromatography, and the possible products in the solution were analyzed by an ion chromatograph. The main products are CO and H_2_ in the headspace. No formate was detected in the liquid phase. The error bars in the plots represent the standard deviations of three independent measurements.

**Determination of QEs for CO production**

A reported method^9^ was used to determine QE. A typical experiment employed a mixture of CoPc (0.1 mM), IrQPY (0.1 mM), TEA (2.5 v%), PhOH (6.0 v%), BIH (80 mM) in 4.0 mL CH_3_CN as the sample solution for evaluation in a cylindrical home-made quartz cell (Supplementary Figure 9, path 1.5 cm). The temperature was kept at 25 °C. The light source is an LED light (*λ* = 450 ± 5 nm, light intensity = 100 mW cm^−2^, irradiated area is 0.8 cm^2^). The photon flux was determined to be 3.01 × 10^-7^ einstein s^-1^.^10^ Under these conditions, the light entering the reaction solution was considered to be fully absorbed by PS, suggesting the evaluated QE is a lower limit. The 1 h of light irradiation is consistent with the total number of photons *n*_p_ = 1.08 × 10^-3^ einstein. The QE was evaluated by the equation S1 for two-electron reduction of CO_2_ with BIH.

QE = *n* (CO) / *n*_p_ (S1)

A typical QE of 10.2% was determined by the measured *n* (CO) = 110.1 *μ*mol by GC-TCD after 1 h of irradiation. In the case of IrQPY/FePor system, the highest QE of 1.7% was determined by the measured *n* (CO) = 71.7 *μ*mol after 4 h of irradiation.

**Determination of quenching rate (*k*_q_)**

A reported method^11^ was followed to calculate the *k*_q_ in the steady-state measurements, where the fluorescence intensity of photo-excited Ir PS in the presence of quencher was measured and fitted to the Stern-Volmer equation,

$\frac{I_{0}}{I}=1+K[Q]=1+k_{q}\tau_{0}[Q]$ (S2)

In this equation, *I*_0_ and *I* are the fluorescence intensity values in the absence and presence of the quencher, *K* is the Stern-Volmer constant for dynamic quenching, *k*_q_ is the apparent rate of bimolecular quenching, *τ*_0_ is the lifetime of the excited state without quencher, and [Q] is the concentration of the quencher.

In the time-resolved fluorescent quenching experiments, the calculation equation should be

$\frac{\tau_{0}}{\tau}=1+k_{q}'\tau_{0}[Q]$ (S3)

Here *k*_q_' is the dynamic quenching rate.

**Determination of second-order reaction rate (*k*_r_)**

A reported method^12^ was followed to calculate the *k*_r_, where the lifetime of reduced Ir PS in the presence of quencher was measured and fitted to the following equation,

$\frac{\tau_{1}}{\tau}=1+k_{r}\tau_{1}[Q]$ (S4)

In this equation, *τ*_1_ and *τ* are the lifetimes in the absence and presence of the quencher, and [Q] is the concentration of the quencher.

**Nanosecond TA spectroscopy**

Nanosecond transient absorption spectra were measured on the LP980 laser flash photolysis instrument (Edinburgh, UK). The pump beam was generated from a tunable laser: Opolette HE 355 LD+UV laser system from OPOTEK (355 nm/410-700 nm, 20/100 Hz, > 4.3 mJ per pulse, and 1 kHz repetition rate). Output pulse of 355 nm from the regenerative amplifier was split into two parts with a beam splitter. The acquisition of transient spectra in this work over a wide wavelength range (300-1000 nm). The scan range for decay was 100 ns (1 ns resolution) to 1000 μs (10 μs resolution), which is optional. After penetrating the sample, the probe beam was collimated to focus into a fiber-coupled spectrometer and detected at a frequency of 1 kHz. The intensity of the pump pulse used in this experiment was controlled by a variable neutral-density filter wheel. The delay between the pump and probe pulses was controlled by a motorized delay stage. The pump pulses were chopped by a synchronized chopper at 500 Hz. The pump pulse was kept in a weak regime where the excitonic annihilation effect can be neglected.

After obtaining the lifetime decay diagram of a certain wavelength, the lifetime was fitted using the Tail Fitting method (Range: start from the top point that upon excited to the base point that contour to before excited; Fitting function: exponential function; Index number: singlet to triplet), and then the Map scan of the whole spectrum at the same excited wavelength is carried out (Map processing method: select the number of bars for equidistant distribution, moderately smoothing).

**Determination of rate constants of electron transfer (*k*_ET_)^13^**

The values of *k*_ET_ were calculated based on the TA data, by multiplying the *k*_r_ with the used quencher concentration ([catalyst] = 0.1 mM), as indicated by equation S5.

$k_{\mathrm{ET}}=k_{r}[\mathrm{catalyst}]$ (S5)

**Supplementary figures**


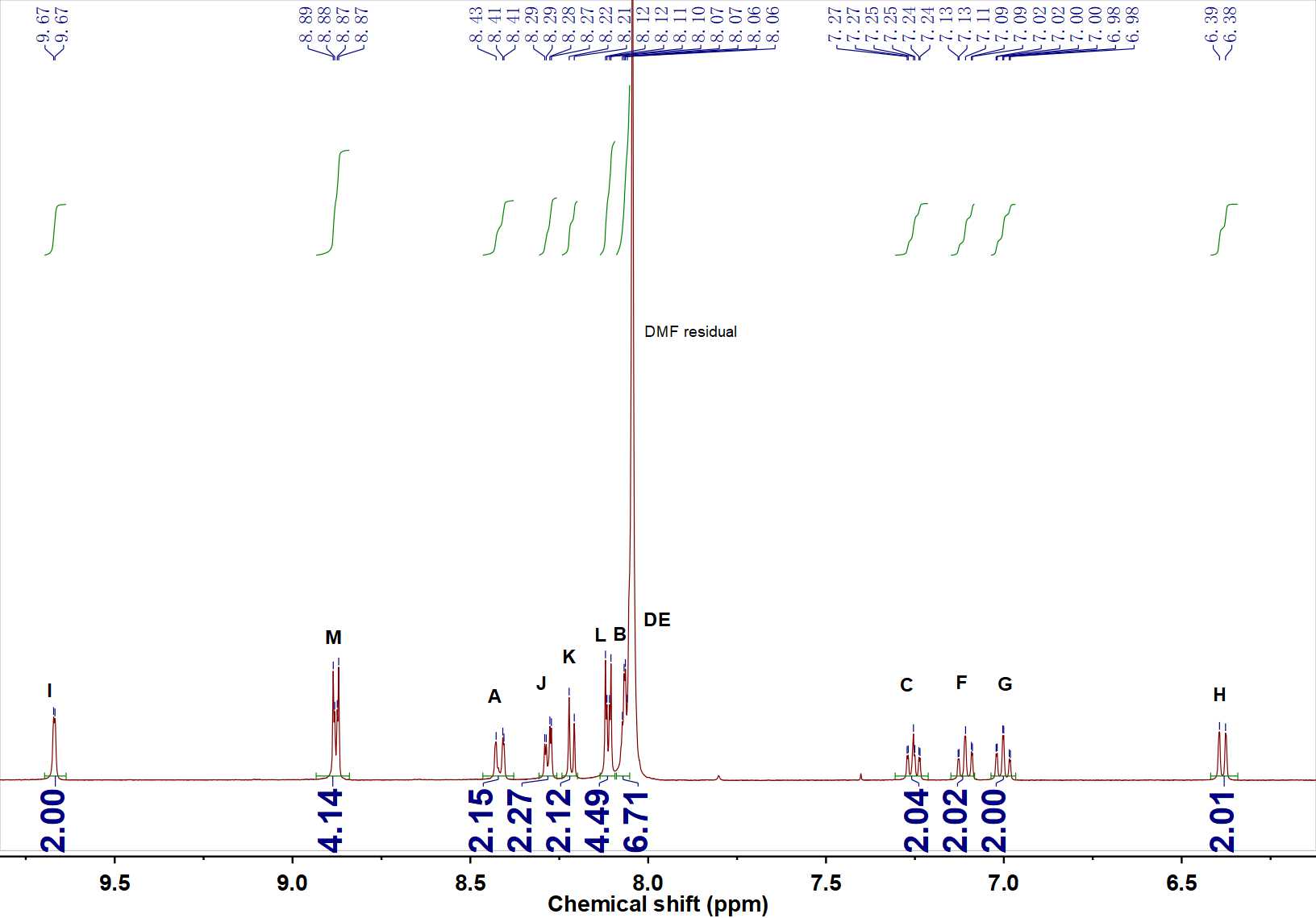


**Supplementary Figure 1 | ^1^H NMR.** ^1^H NMR spectrum of a 2.0 mM DMF-d_7_ solution of IrQPY, with assignment of proton positions in cooperation with the subsequent 2D COSY results in Supplementary Figure 3. Protons D and E are eluded by the DMF solvent peak, which will not interfere the titration analysis that only considers the protons (I, J, K, L and M) from the qpy ligand.


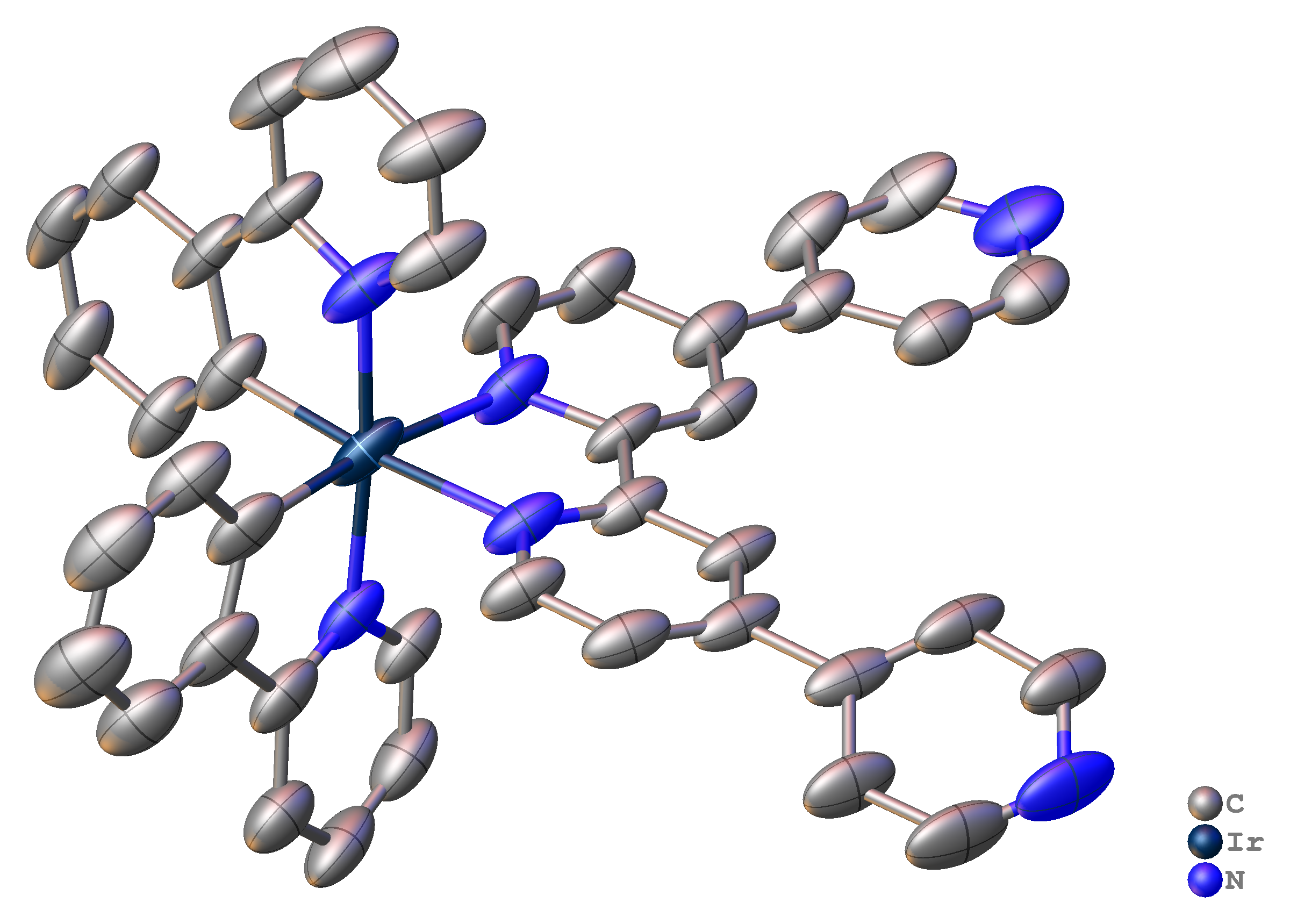


**Supplementary Figure 2 | Crystal structure.** Crystal structure of the cation of IrQPY. Counter anions and solvent molecules are omitted for clarity. Possibility = 50%


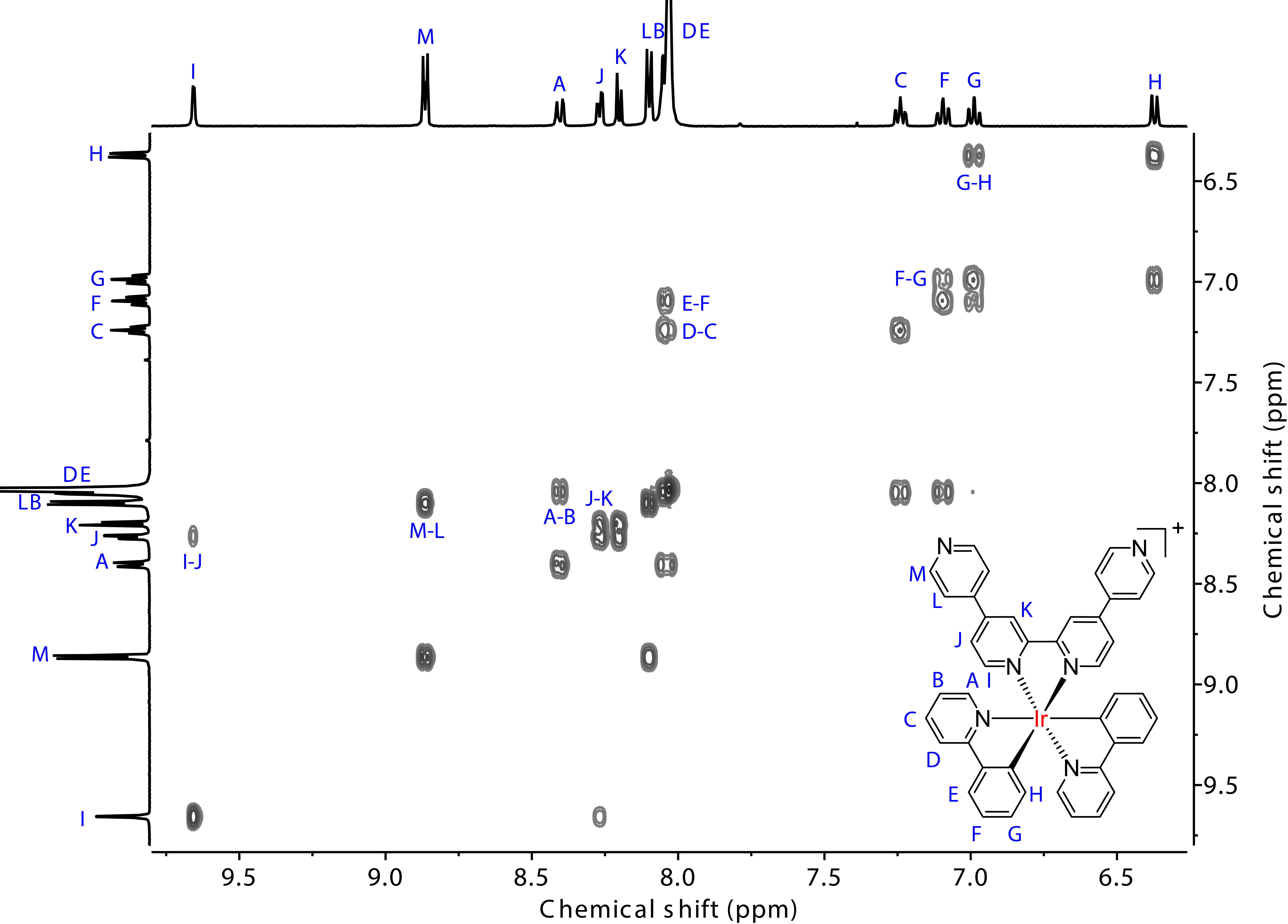


**Supplementary Figure 3 |** **^1^H-^1^H 2D COSY NMR.** ^1^H-^1^H 2D COSY NMR spectrum of a 2.0 mM DMF-d_7_ solution of IrQPY. Protons D and E are eluded by the DMF solvent peak, which will not interfere the titration analysis that only considers the protons (I, J, K, L and M) on the qpy ligand. The equivalent protons are not labelled for clarity.


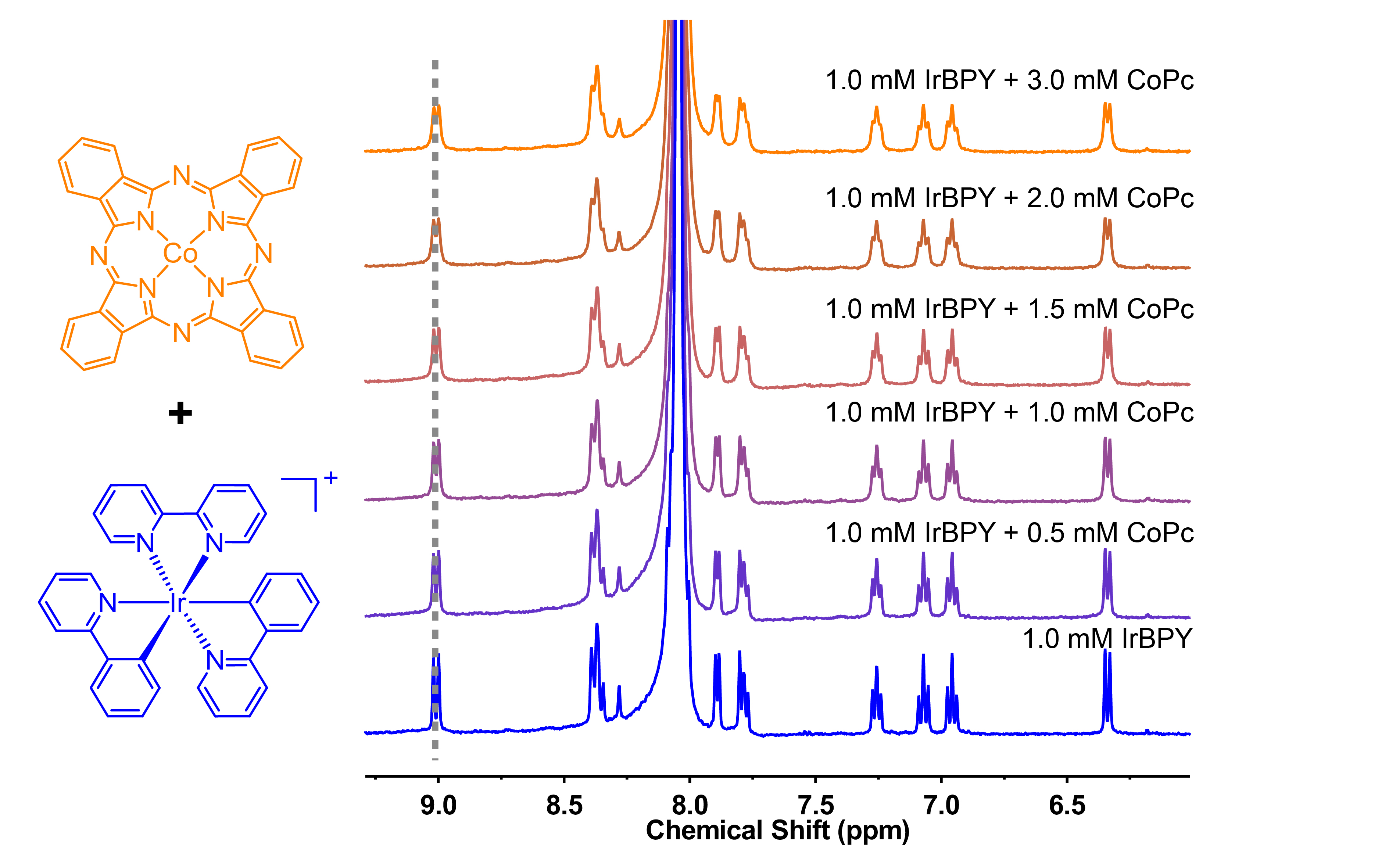


**Supplementary Figure 4 | ^1^H NMR titration.** ^1^H NMR titration of CoPc into a 1.0 mM DMF-d_7_ solution of IrBPY.


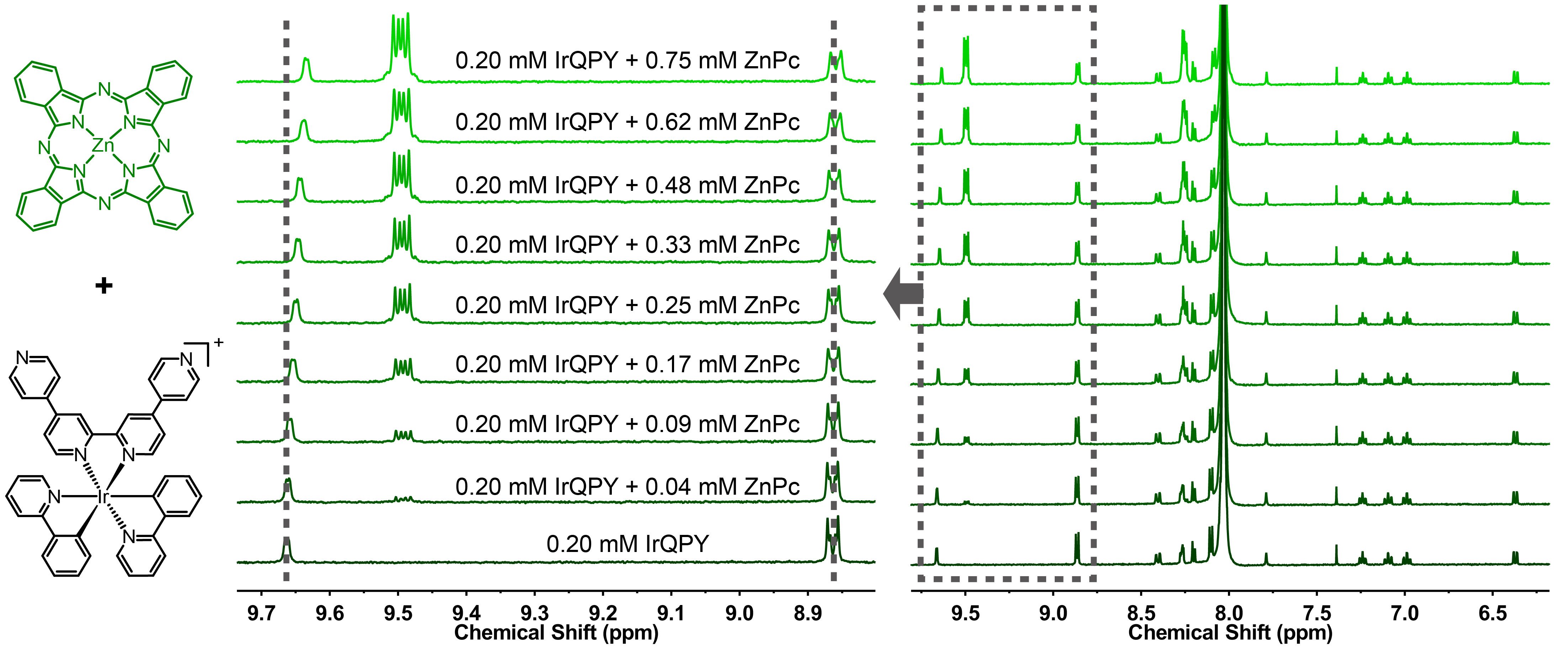


**Supplementary Figure 5 | ^1^H NMR titration.** ^1^H NMR titration of ZnPc into a 0.20 mM DMF-d_7_ solution of IrQPY.





**Supplementary Figure 6 | Cyclic voltammetry.** CVs of 0.5 mM CoPc under N_2_ (black), under CO_2_ (brown) or added 6 v% PhOH under CO_2_ (orange) in CH_3_CN.


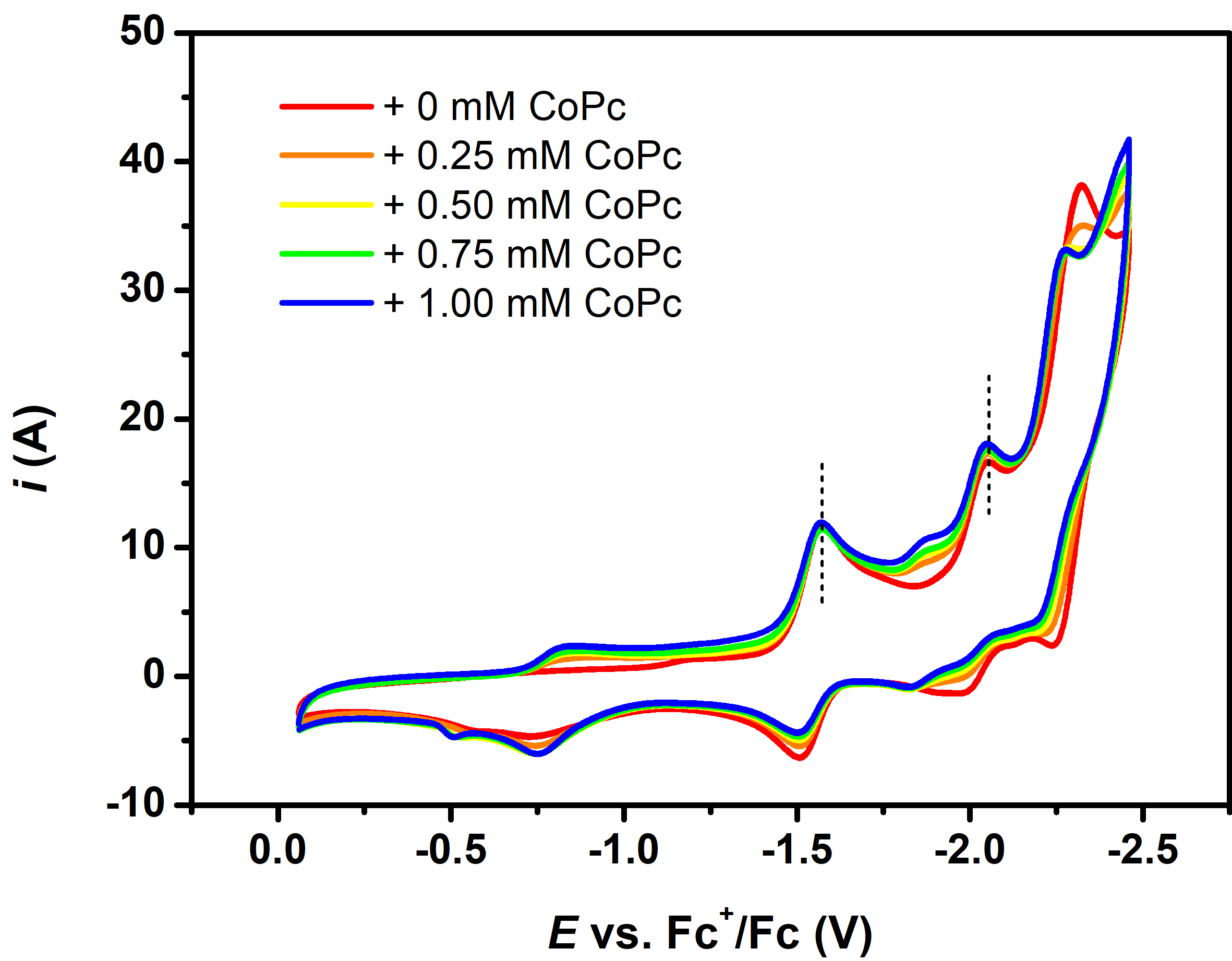


**Supplementary Figure 7 | Cyclic voltammetry.** CV of 0.5 mM IrQPY in dry CH_3_CN under N_2_ without or with increasing concentration of CoPc (0~1.0 mM).


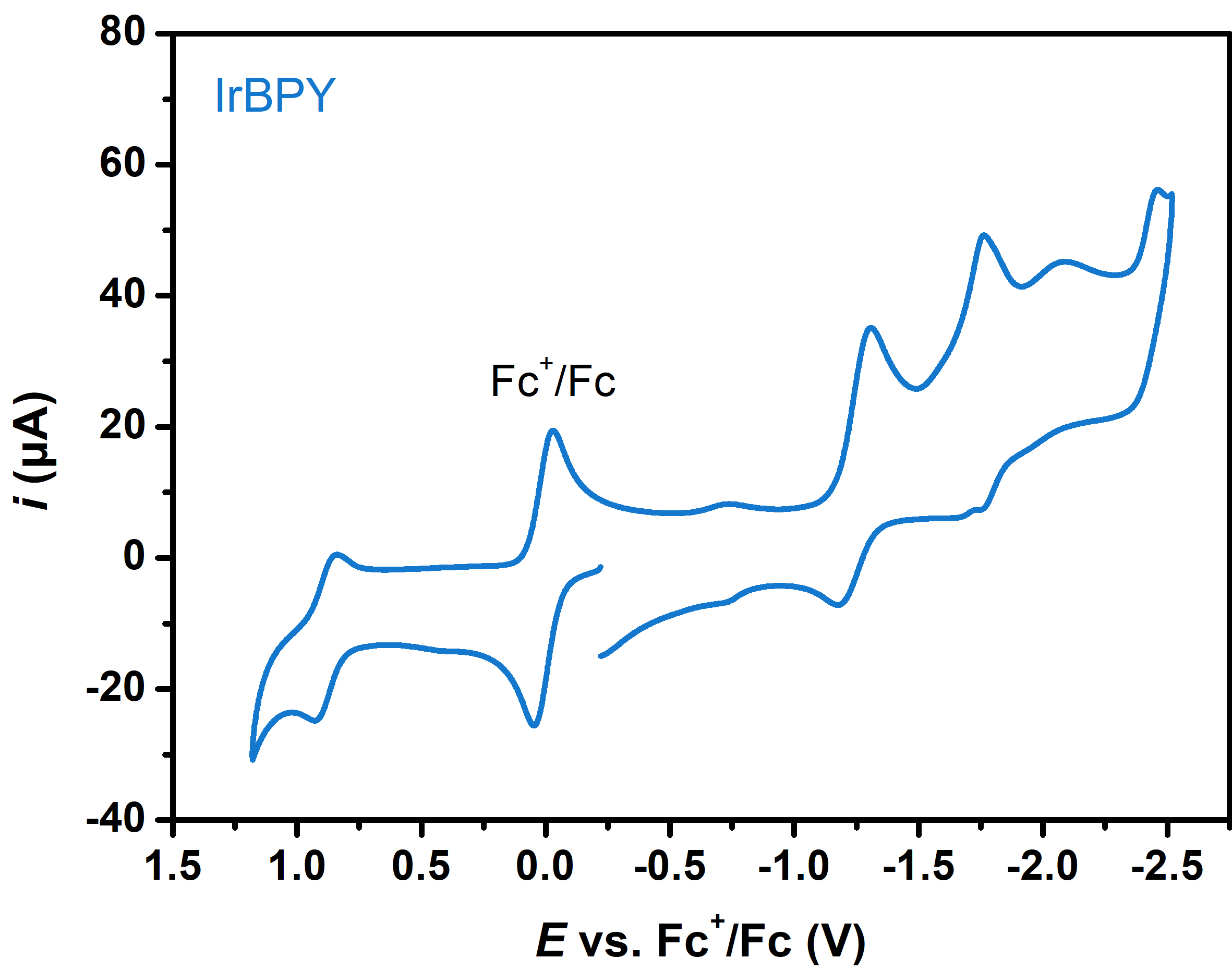


**Supplementary Figure 8 | Cyclic voltammetry.** CV of 0.5 mM IrBPY in dry CH_3_CN under N_2_.


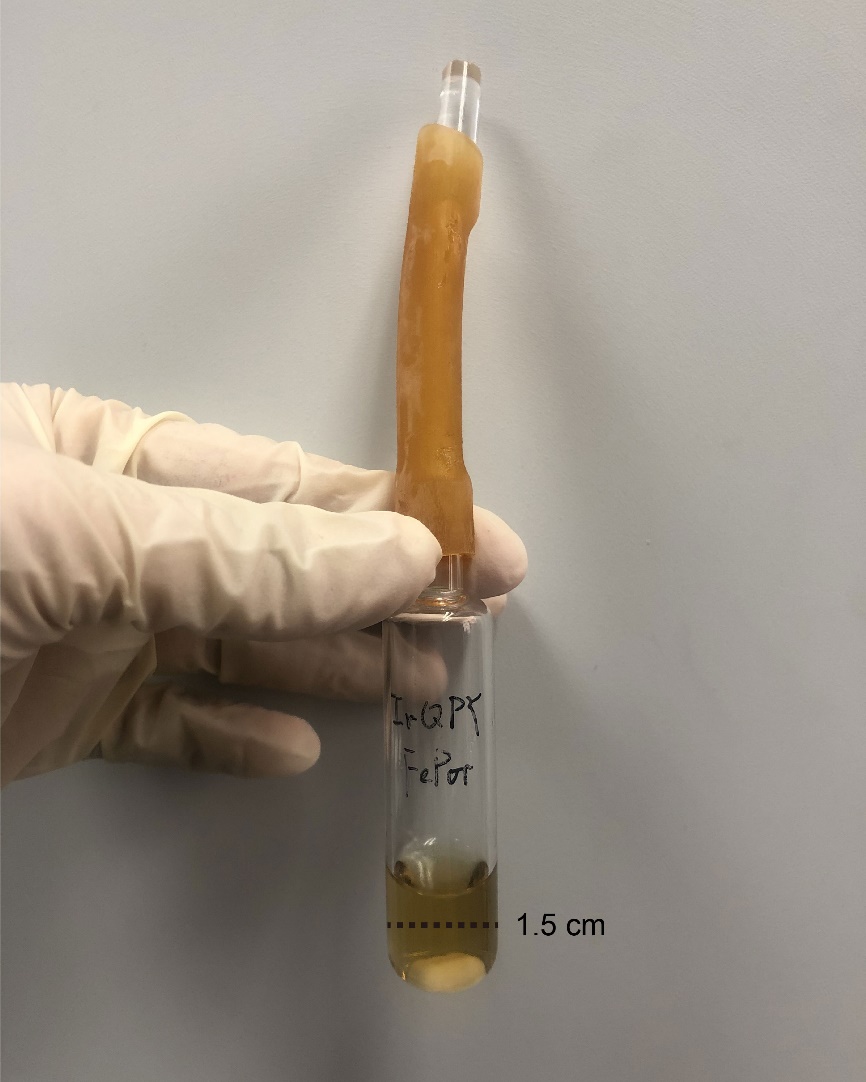


**Supplementary Figure 9 | Photocatalytic set-up.** Photo of the used home-made quartz cell for light-driven photocatlaytic CO_2_ reduction.


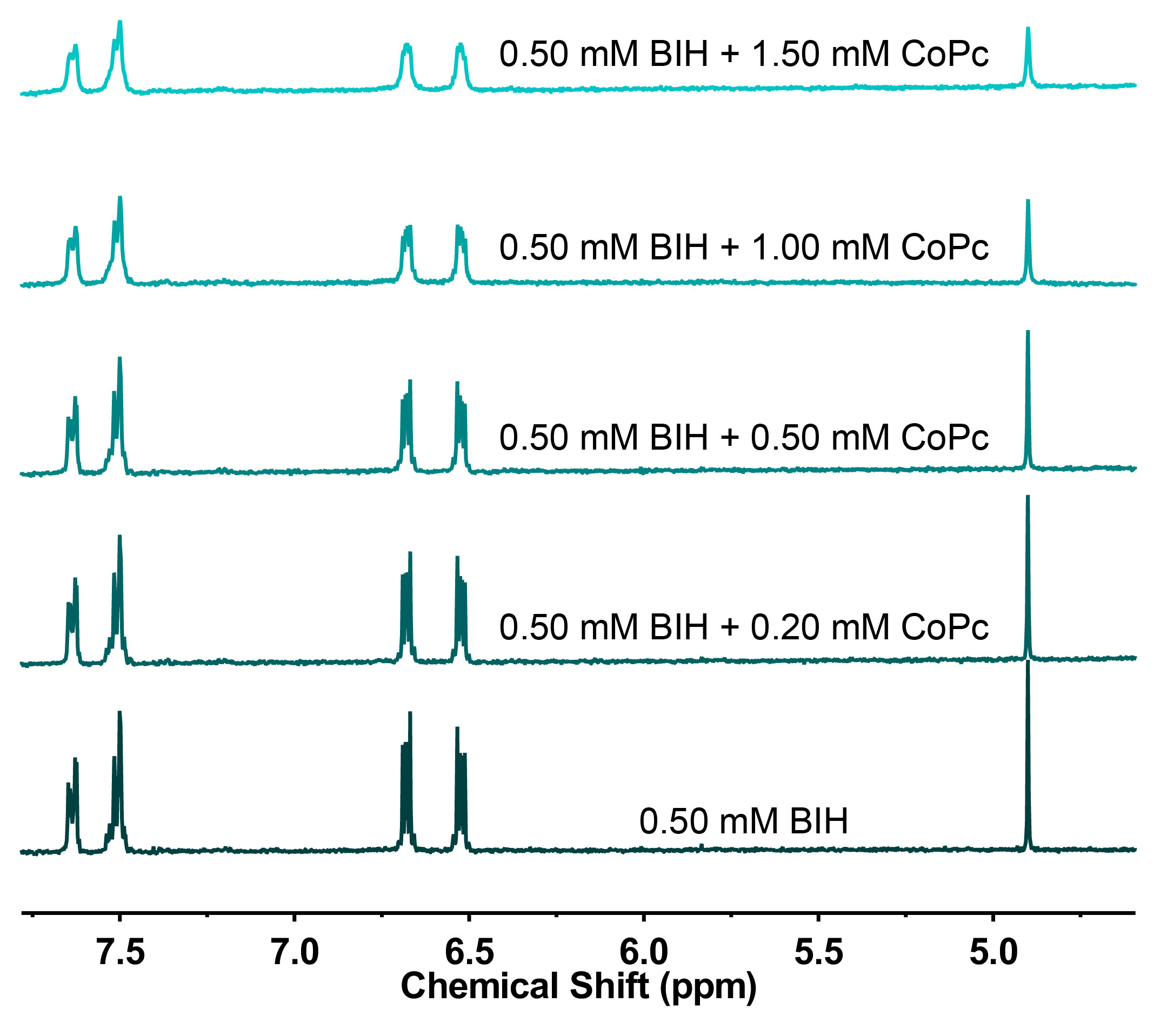


**Supplementary Figure 10 | ^1^H NMR titration.** ^1^H NMR titration of CoPc into a 0.50 mM DMF-d_7_ solution of BIH.


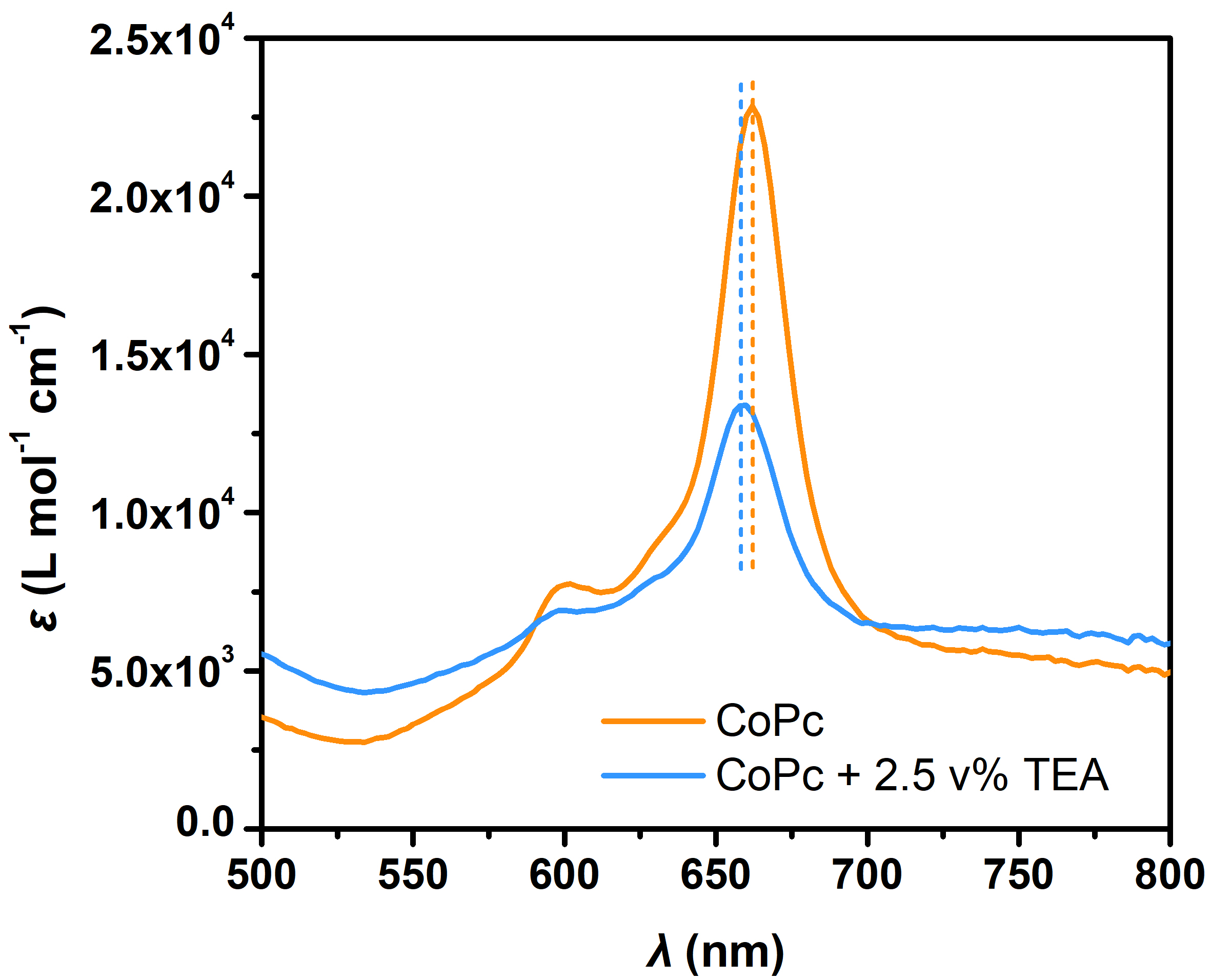


**Supplementary Figure 11 | UV-Vis spectroscopy.** UV-Vis spectra of 10 μM CH_3_CN solution of CoPc in the absence of presence of 2.5 v% TEA.


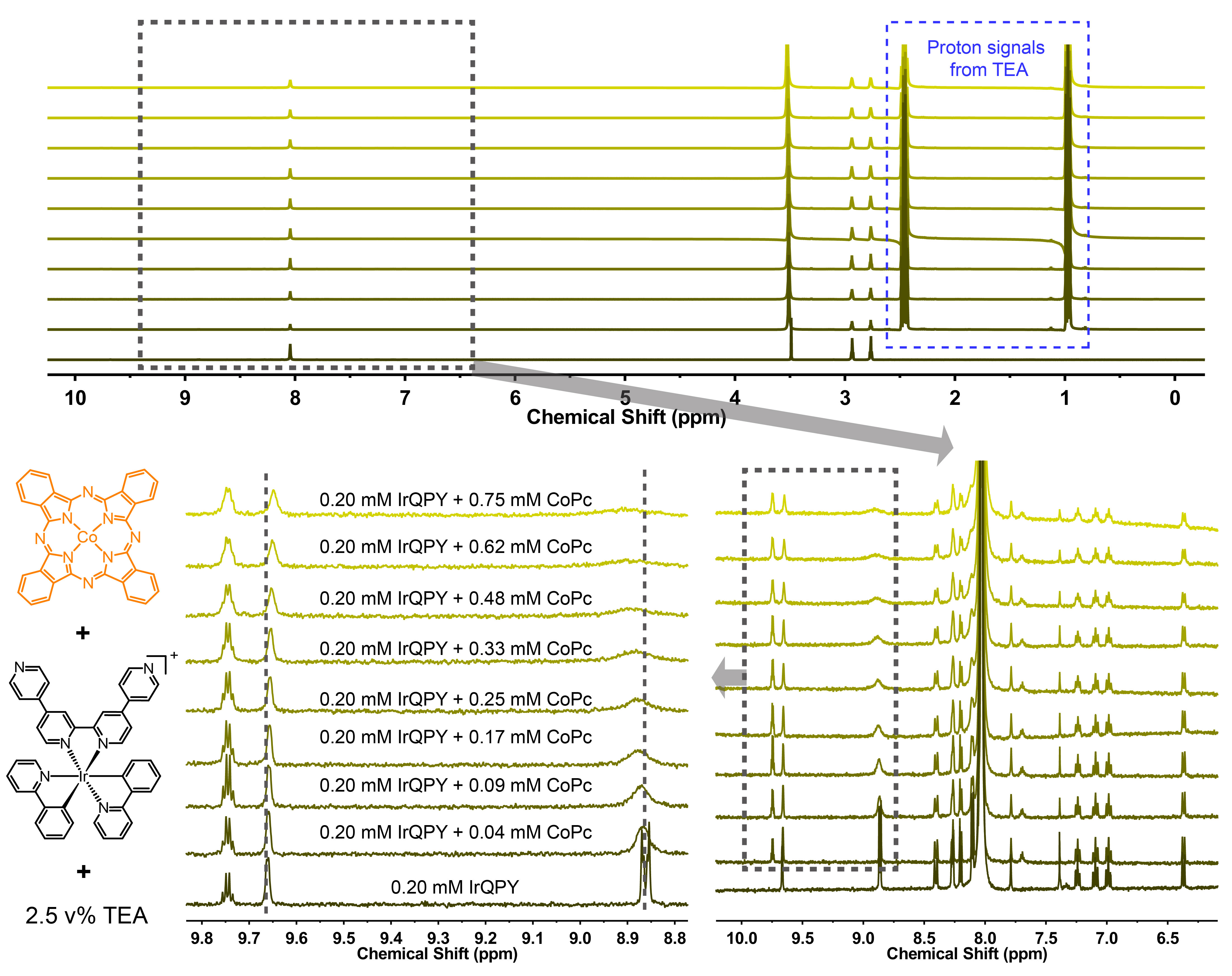


**Supplementary Figure 12 | ^1^H NMR titration.** ^1^H NMR titration of CoPc into a 0.20 mM DMF-d_7_ solution of IrQPY in the presence of 2.5 v% TEA. The proton signals of protonated TEA locate at ca. 9.76 ppm, and the protonation should be caused by the remnant water.


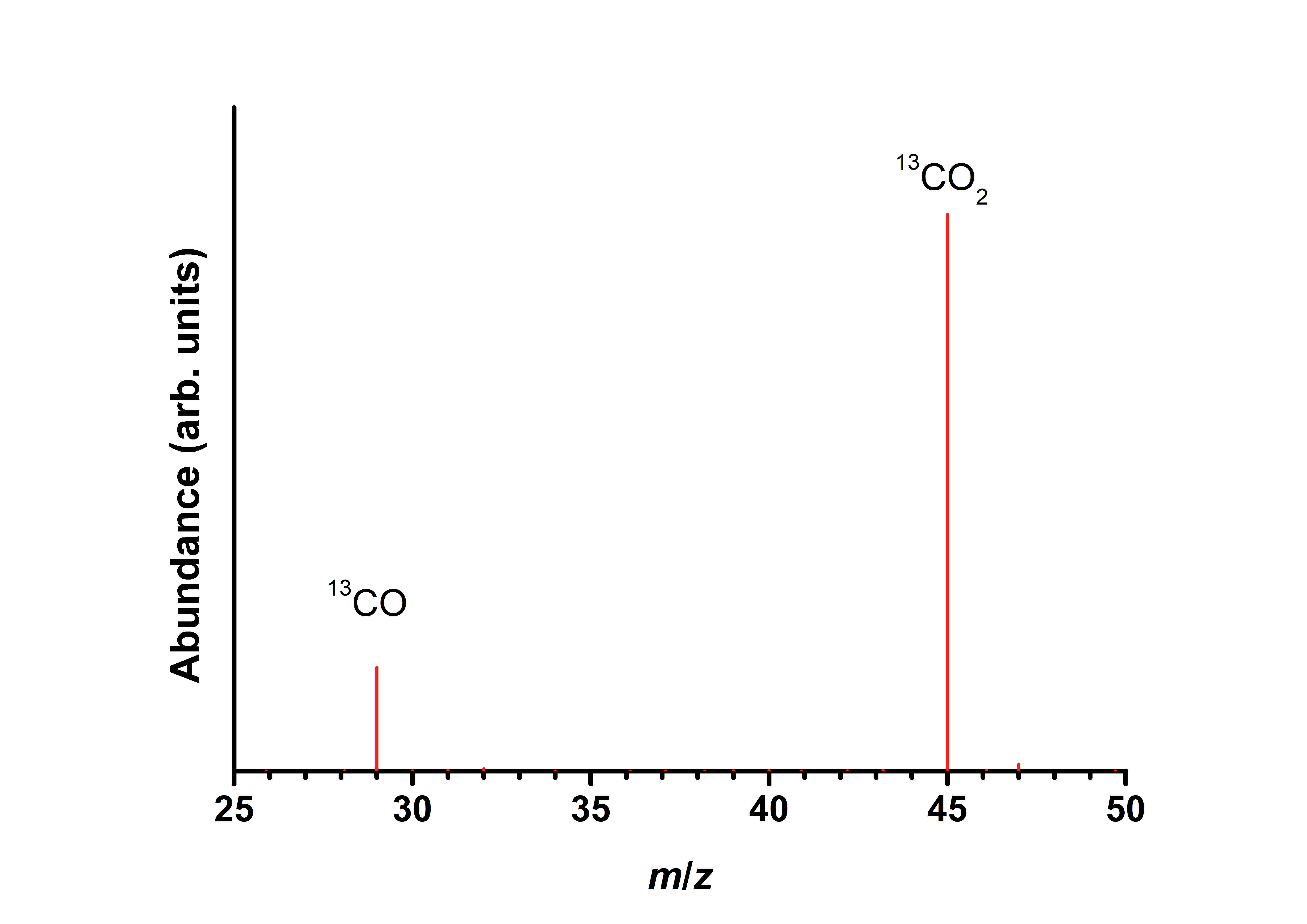


**Supplementary Figure 13 | Isotope labelling experiment.** Mass spectra via gas chromatography on the generated gas from a mixture of CoPc (0.1 mM), IrQPY (0.1 mM), TEA (2.5 v%), PhOH (6.0 v%), and BIH (80 mM) in 4.0 mL CH_3_CN within 4 h of 450 nm irradiation under 1 atm ^13^CO_2_.


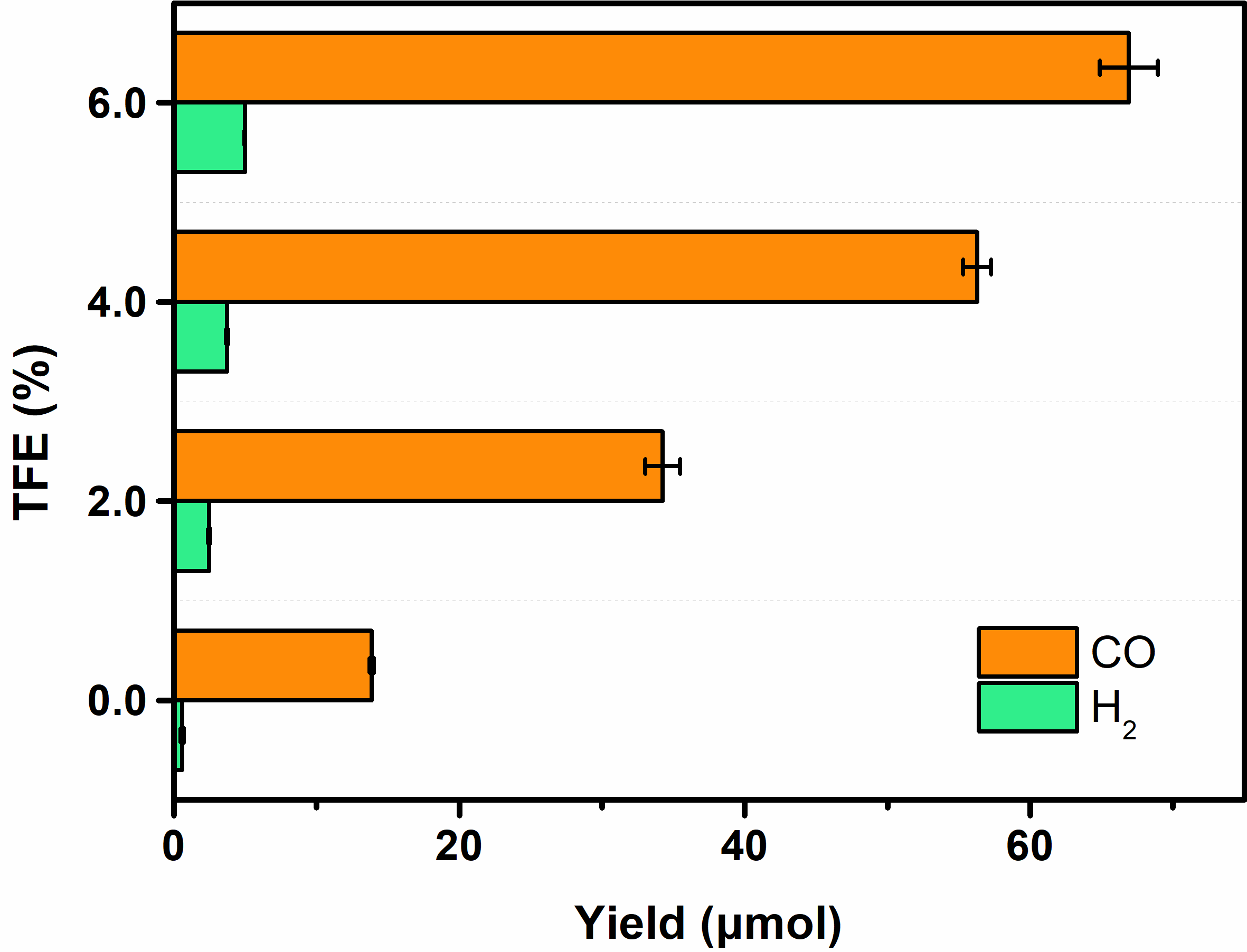


**Supplementary Figure 14 | Photocatalytic CO_2_ reduction.** Photocatalytic CO and H_2_ yields from a mixture of CoPc (0.1 mM), IrQPY (0.1 mM), TEA (2.5 v%), TFE (0, 2.0, 4.0 or 6.0 v%), and BIH (80 mM) in 4.0 mL CO_2_-saturated CH_3_CN within 4 h of 450 nm irradiation under 1 atm CO_2_.


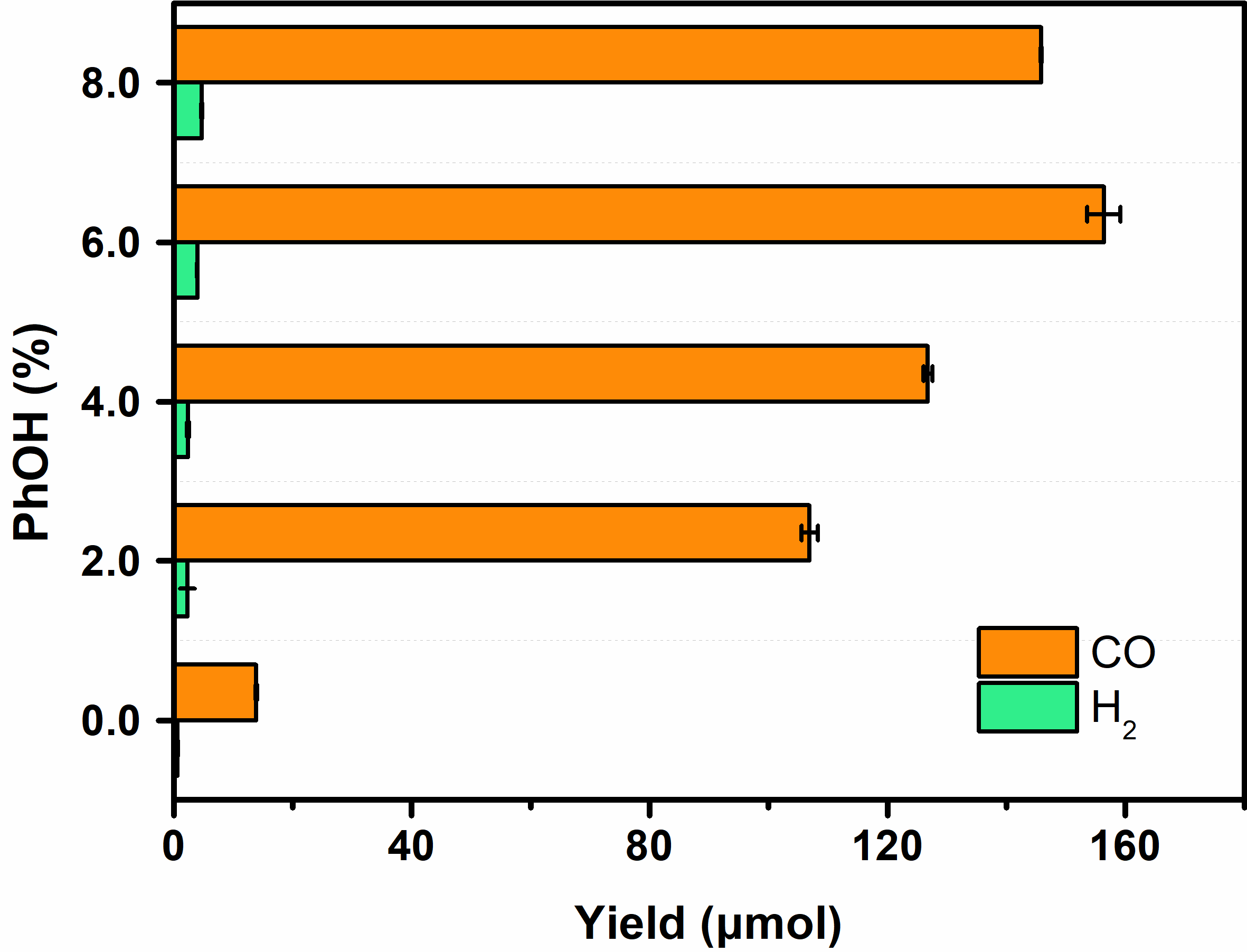


**Supplementary Figure 15 | Photocatalytic CO_2_ reduction.** Photocatalytic CO and H_2_ yields from a mixture of CoPc (0.1 mM), IrQPY (0.1 mM), TEA (2.5 v%), PhOH (0, 2.0, 4.0, 6.0 or 8.0 v%), and BIH (80 mM) in 4.0 mL CH_3_CN within 4 h of 450 nm irradiation under 1 atm CO_2_.


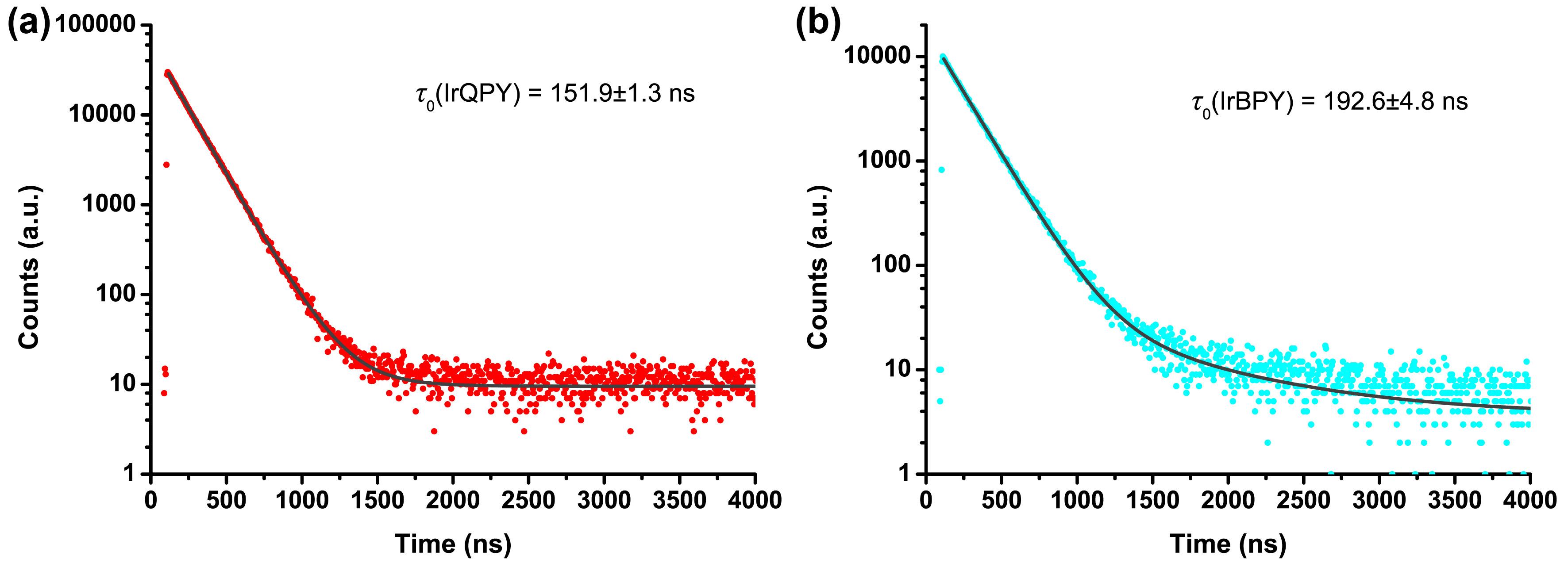


**Supplementary Figure 16 | Excited state lifetimes.** Fluorescence decays of 50 μM **a** IrQPY and **b** IrBPY in CH_3_CN under N_2_.


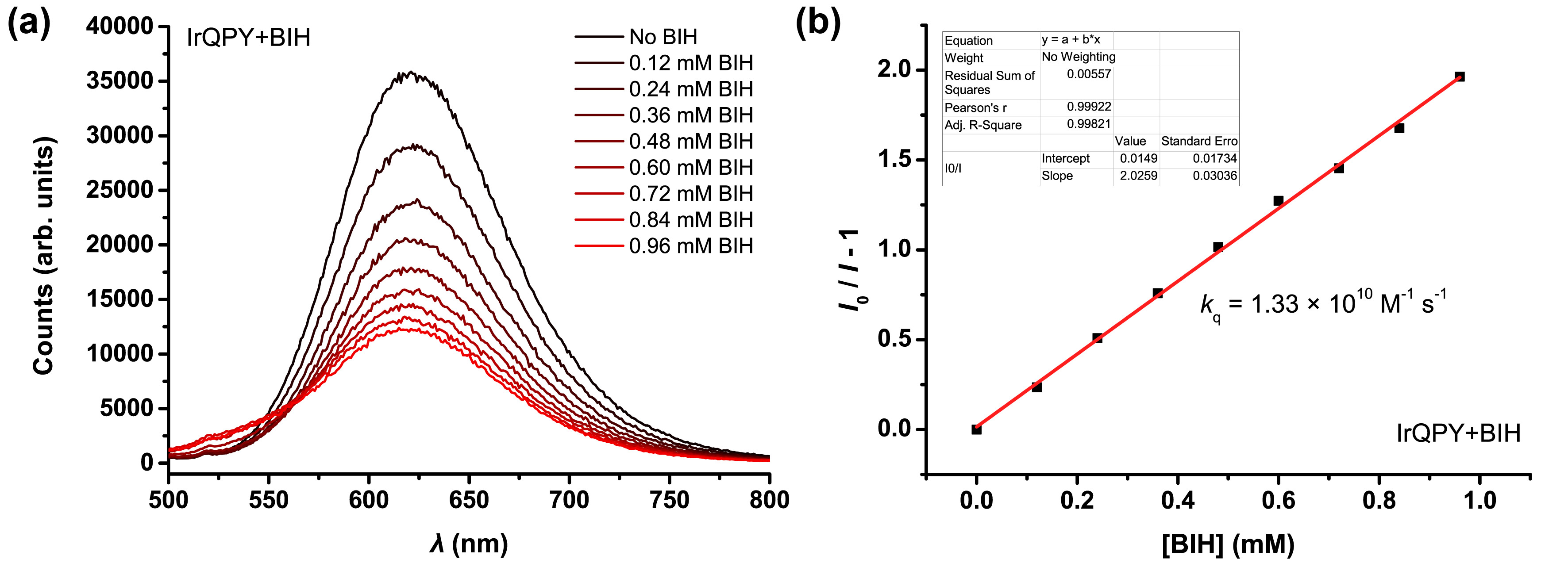


**Supplementary Figure 17 | Quenching experiments.** **a** Fluorescence spectra of a CH_3_CN solution containing 0.05 mM IrQPY in the presence of 0~0.96 mM BIH, respectively. **b** Linear fitting of ratio of fluorescence intensity versus [BIH].


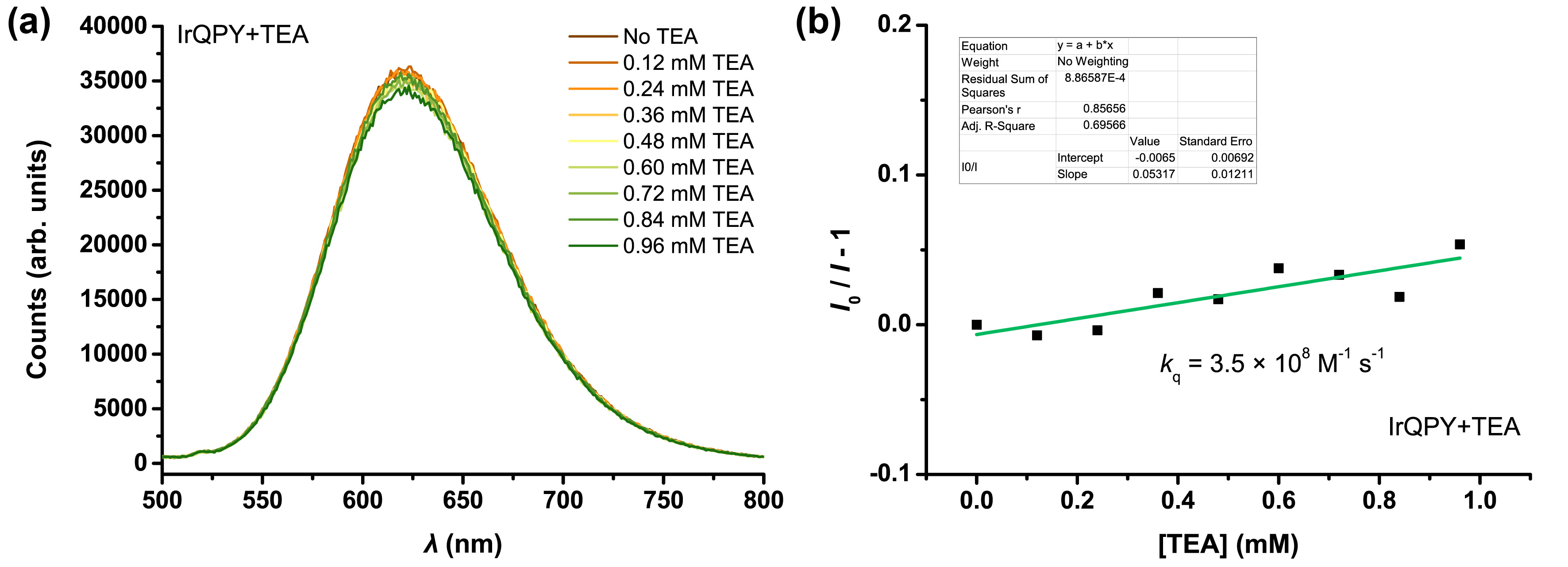


**Supplementary Figure 18** **| Quenching experiments.** **a** Fluorescence spectra of a CH_3_CN solution containing 0.05 mM IrQPY in the presence of 0~0.96 mM TEA, respectively. **b** Linear fitting of ratio of fluorescence intensity versus [TEA].


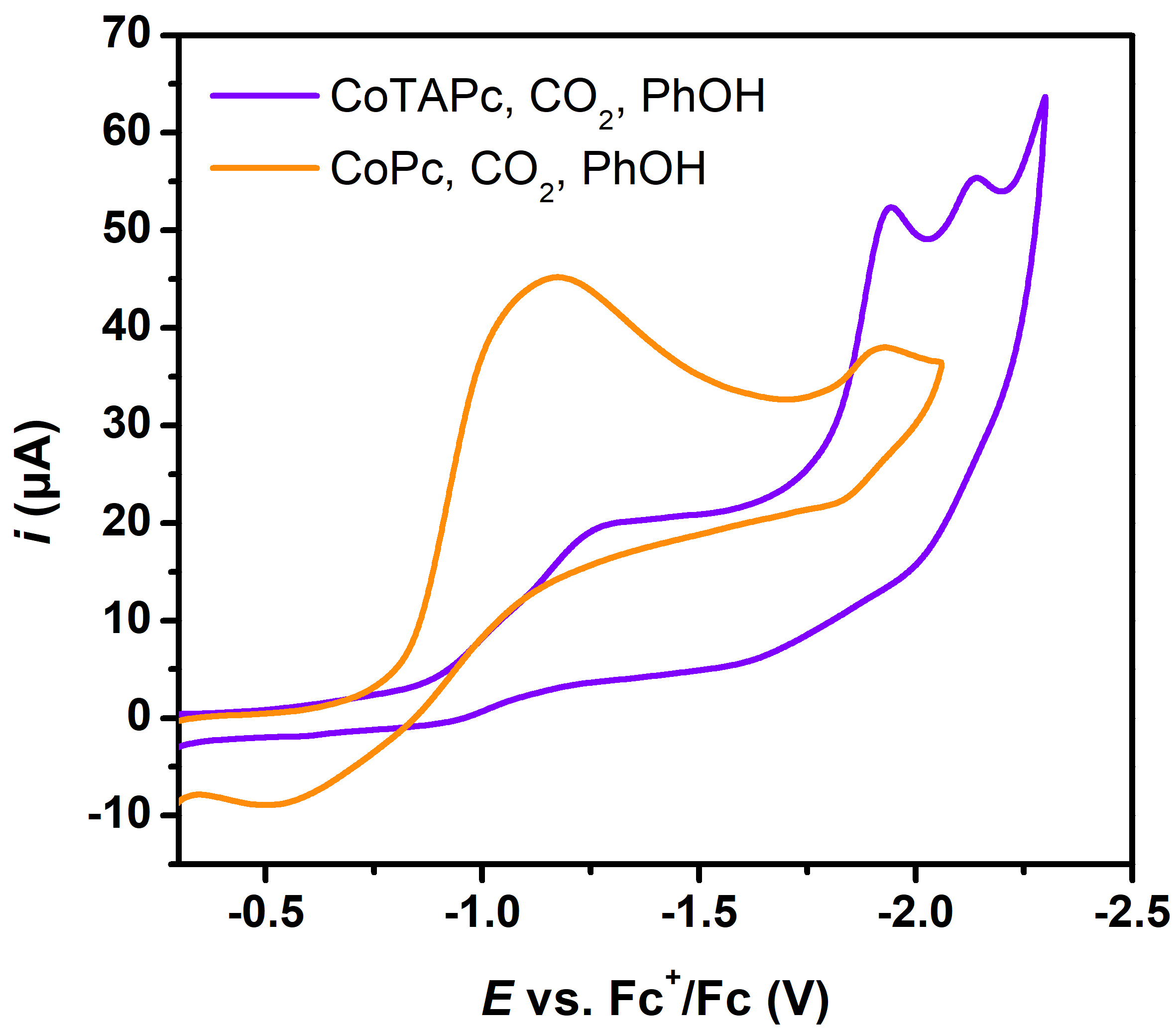


**Supplementary Figure 19 | Cyclic voltammetry.** CVs of CoPc (orange) and CoTAPc (violet) with addition of 6 v% PhOH under CO_2_.


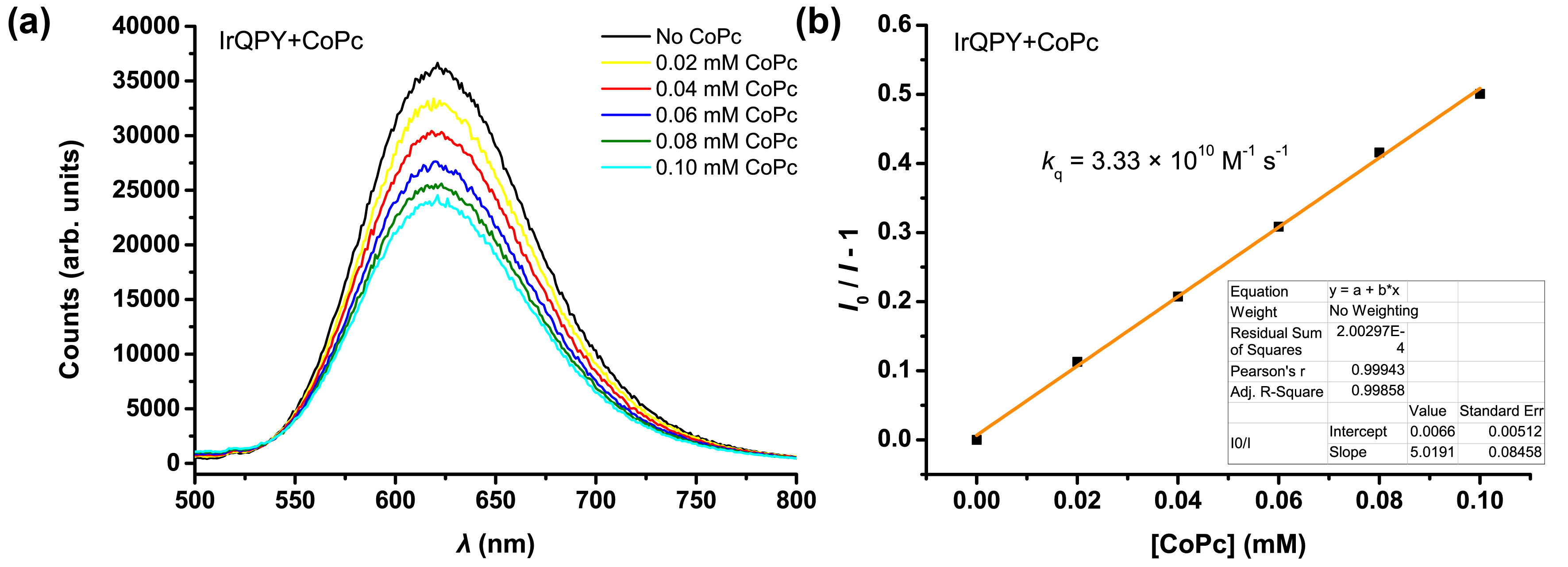


**Supplementary Figure 20 | Quenching experiments.** **a** Fluorescence spectra of a CH_3_CN solution containing 0.05 mM IrQPY in the presence of 0~0.10 mM CoPc, respectively. **b** Linear fitting of ratio of fluorescence intensity versus [CoPc].


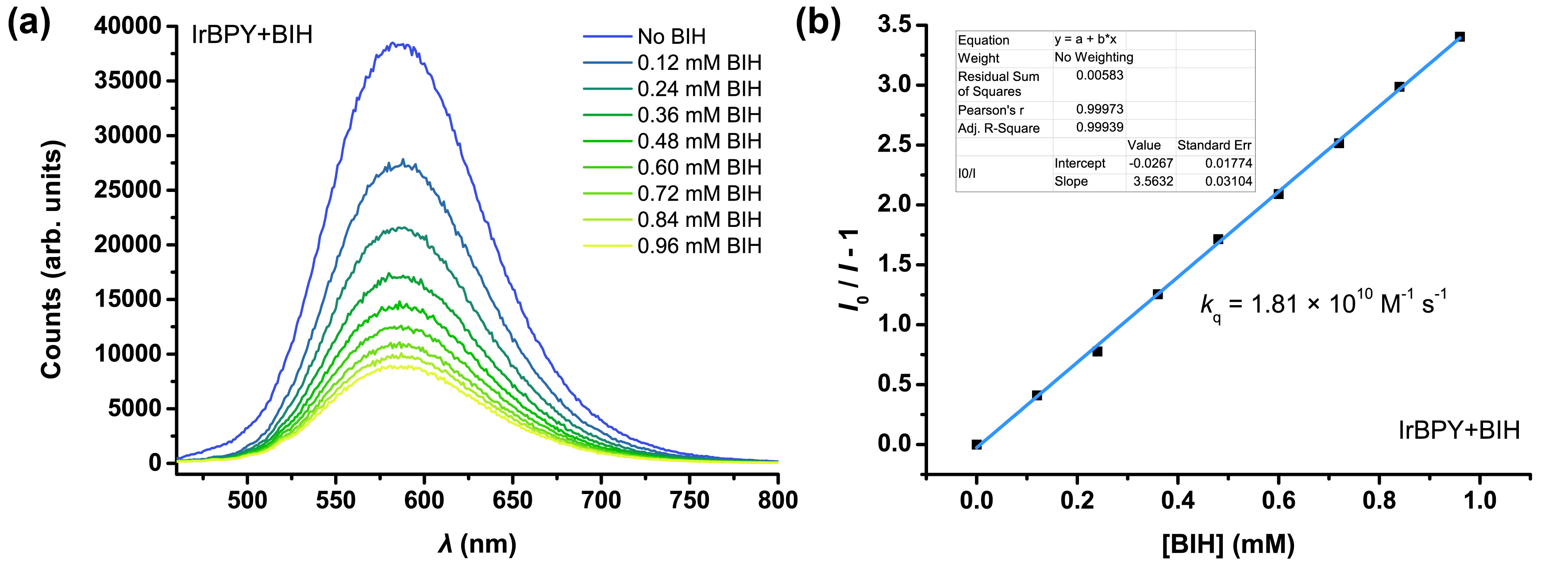


**Supplementary Figure 21 | Quenching experiments.** **a** Fluorescence spectra of a CH_3_CN solution containing 0.05 mM IrBPY in the presence of 0~0.96 mM BIH, respectively. **b** Linear fitting of ratio of fluorescence intensity versus [BIH].


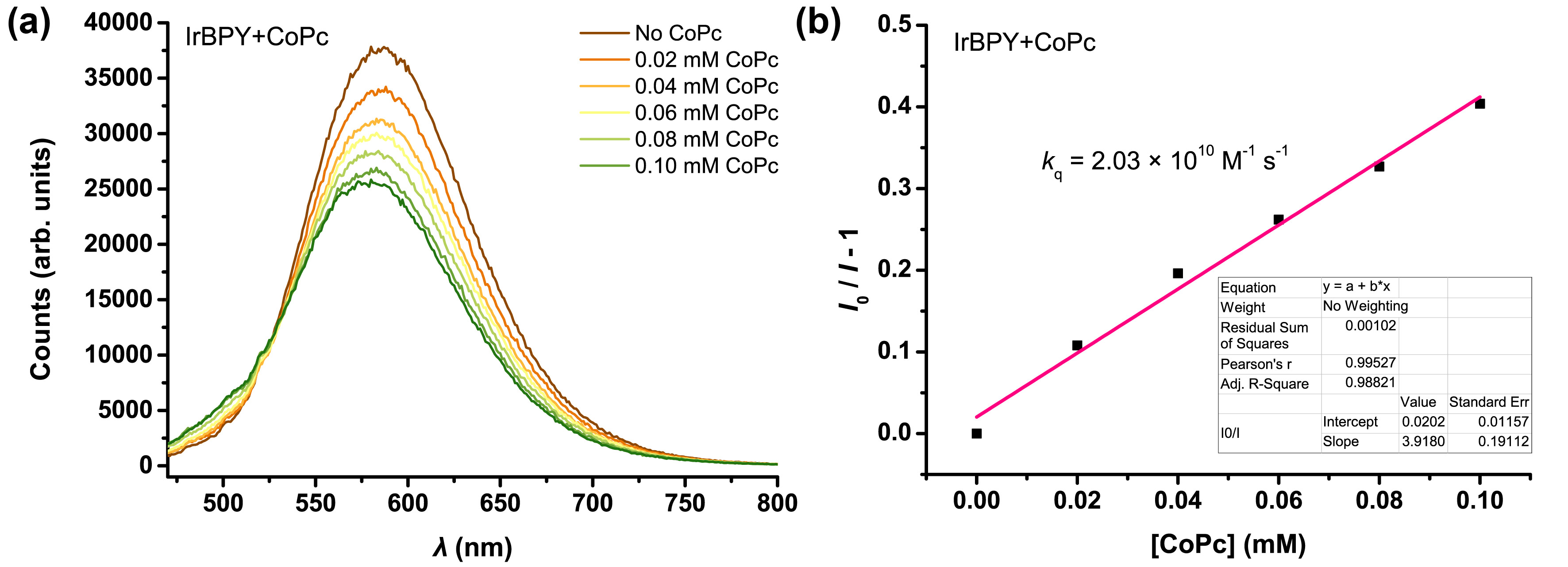


**Supplementary Figure 22 | Quenching experiments.** **a** Fluorescence spectra of a CH_3_CN solution containing 0.05 mM IrBPY in the presence of 0~0.10 mM CoPc, respectively. **b** Linear fitting of ratio of fluorescence intensity versus [CoPc].


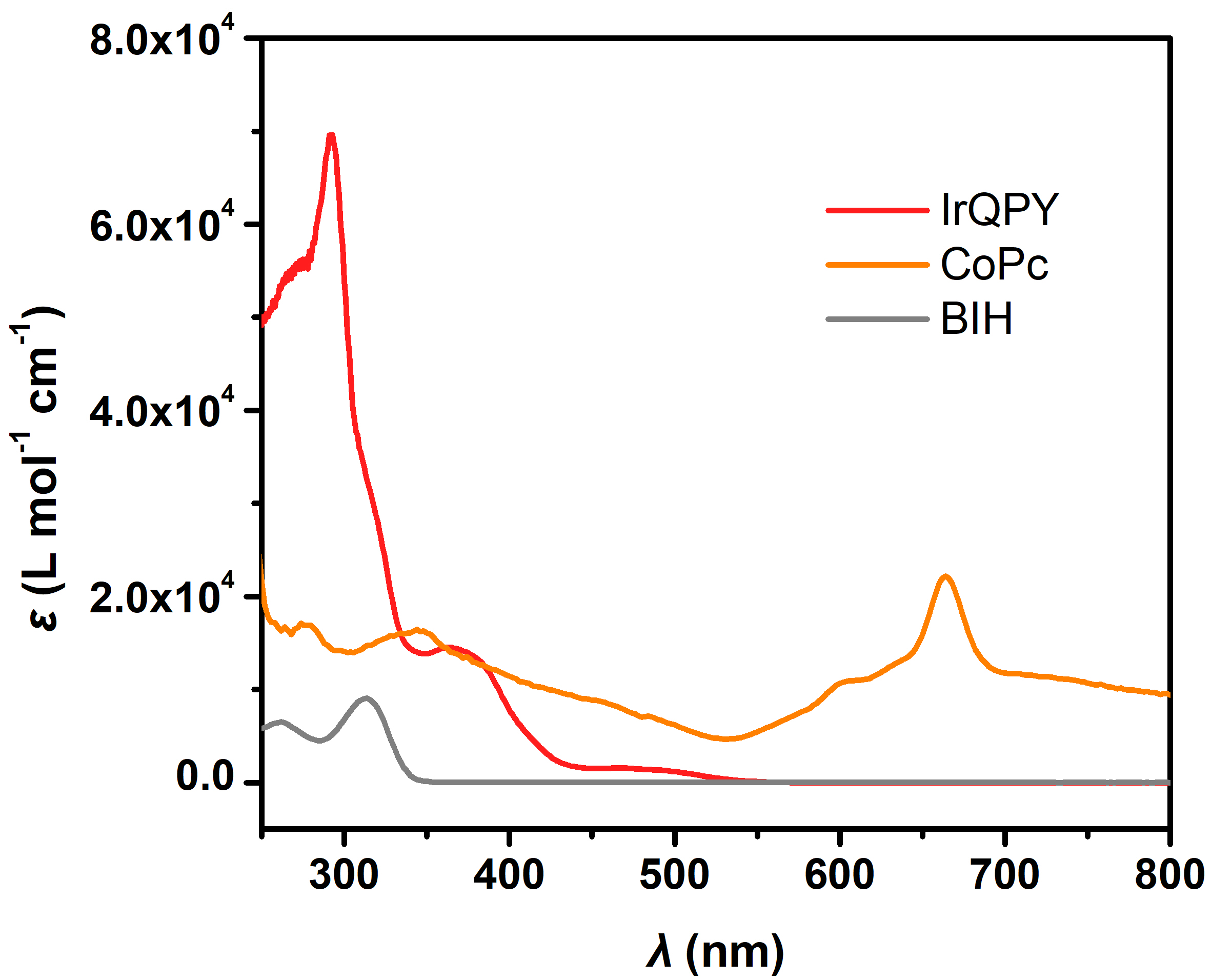


**Supplementary Figure 23 | UV-Vis spectroscopy.** UV-Vis spectra of 50 μM IrQPY (red), 10 μM CoPc (orange) and 10 μM BIH (gray) in CH_3_CN.


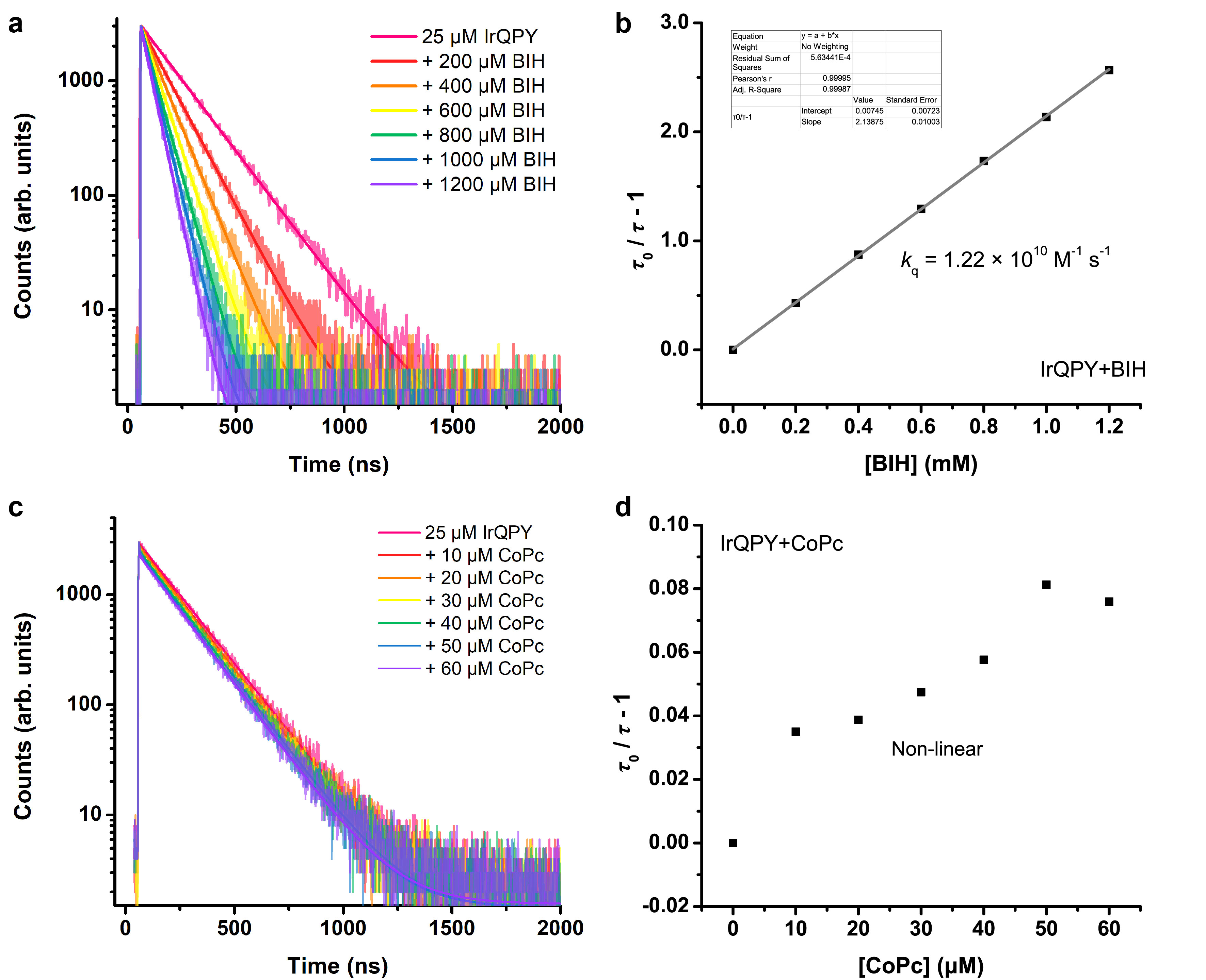


**Supplementary Figure 24 | Time-resolved fluorescent quenching experiments.** **a** Time-resolved fluorescence decay traces of a CH_3_CN solution containing 0.025 mM IrQPY in the presence of 0~1.2 mM BIH, respectively. **b** Linear fitting of ratio of fluorescence lifetime versus [BIH]. **c** Time-resolved fluorescence decay traces of a CH_3_CN solution containing 0.025 mM IrQPY in the presence of 0~60 μM CoPc, respectively. **d** Linear fitting of ratio of fluorescence lifetime versus [CoPc]. Excitation laser is 445.6 nm.


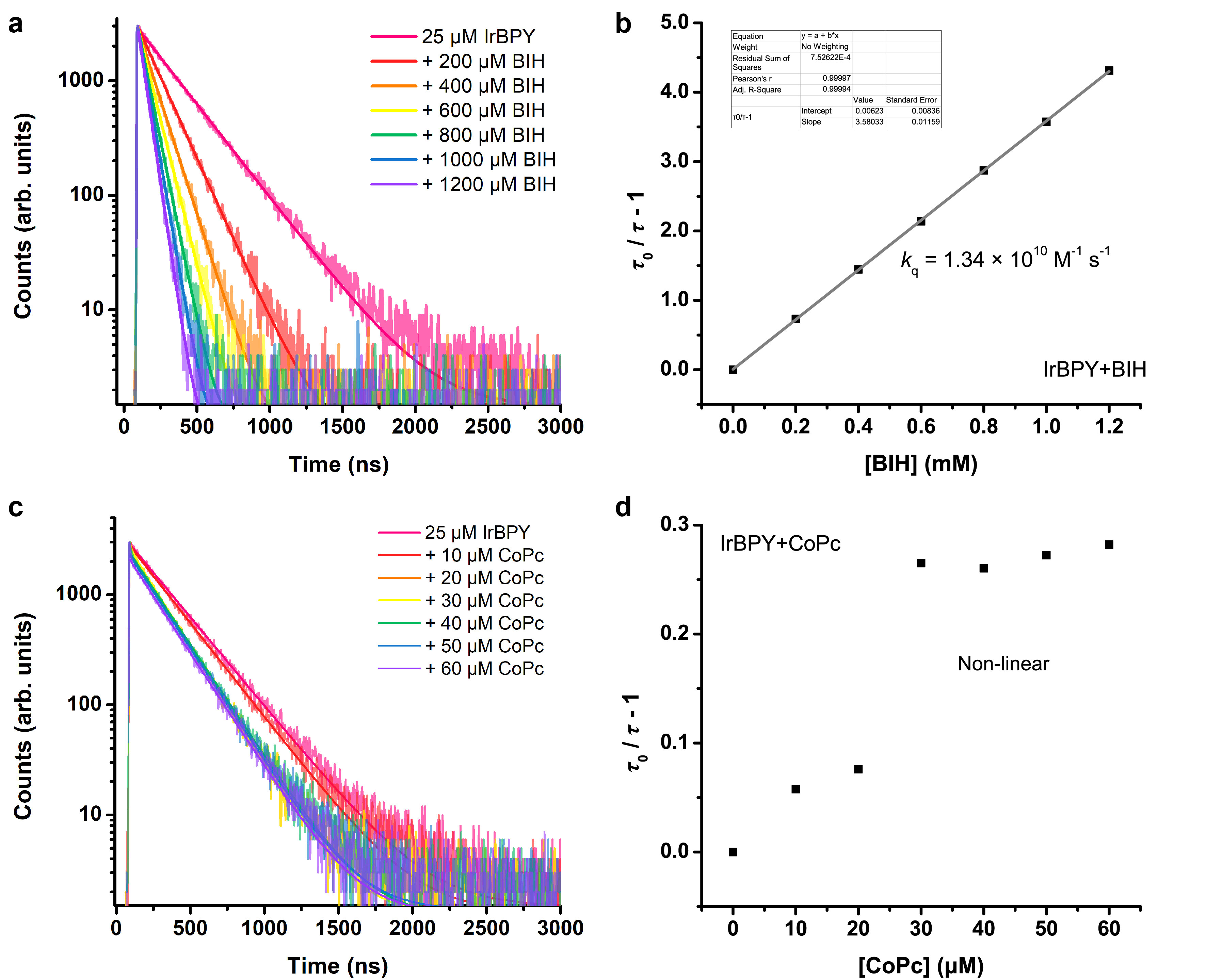


**Supplementary Figure 25 |** **Time-resolved fluorescent quenching experiments.** **a** Time-resolved fluorescence decay traces of a CH_3_CN solution containing 0.025 mM IrBPY in the presence of 0~1.2 mM BIH, respectively. **b** Linear fitting of ratio of fluorescence lifetime versus [BIH]. **c** Time-resolved fluorescence decay traces of a CH_3_CN solution containing 0.025 mM IrBPY in the presence of 0~60 μM CoPc, respectively. **d** Linear fitting of ratio of fluorescence lifetime versus [CoPc]. Excitation laser is 445.6 nm.


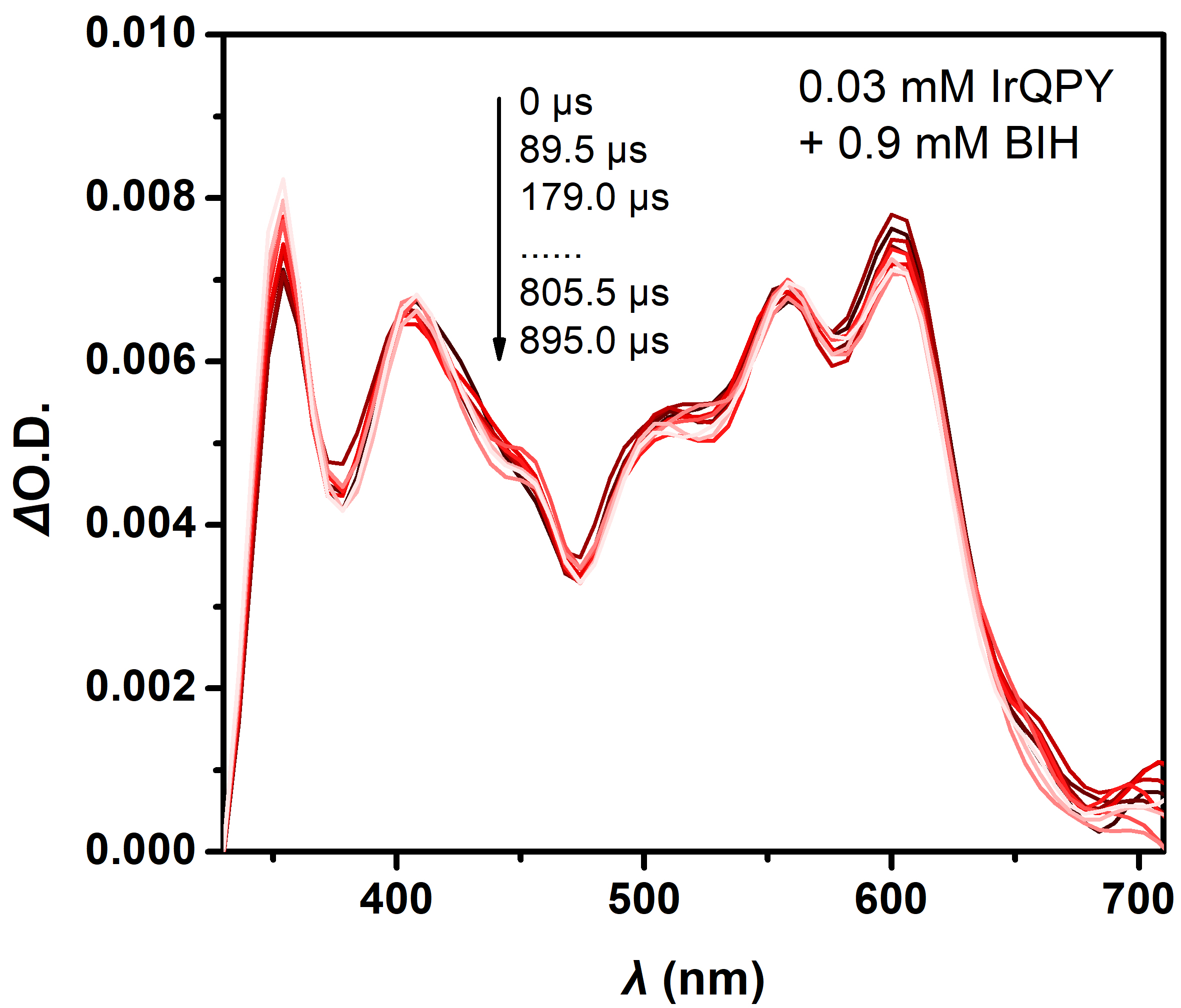


**Supplementary Figure 26** **| Transient absorption spectroscopy**. TA spectrum of 0.03 mM IrQPY in the presence of 0.9 mM BIH.


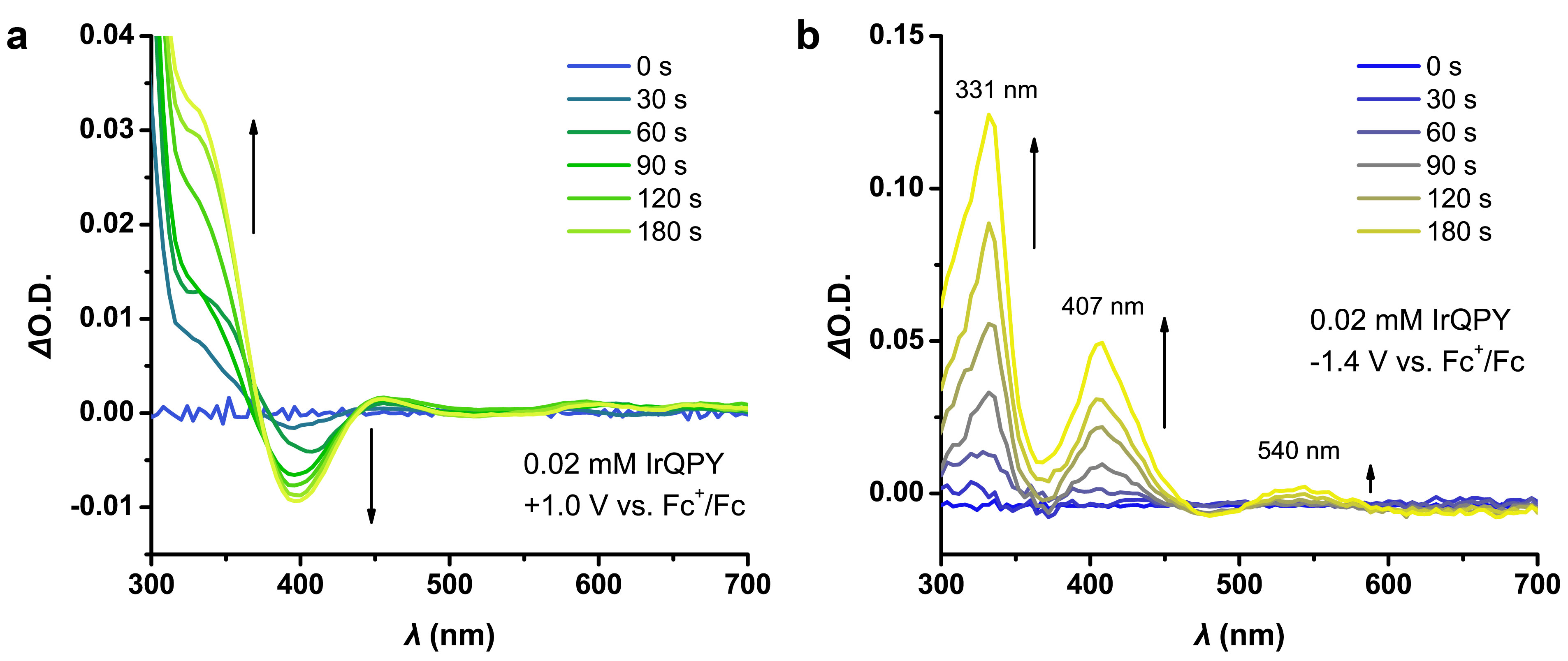


**Supplementary Figure 27** **| Spectroelectrochemistry**. Differential spectra of UV-vis absorption of 0.02 mM IrQPY in the presence of 0.1 M *n*Bu_4_NPF_6_ upon (a) oxidation and (b) reduction under N_2_.


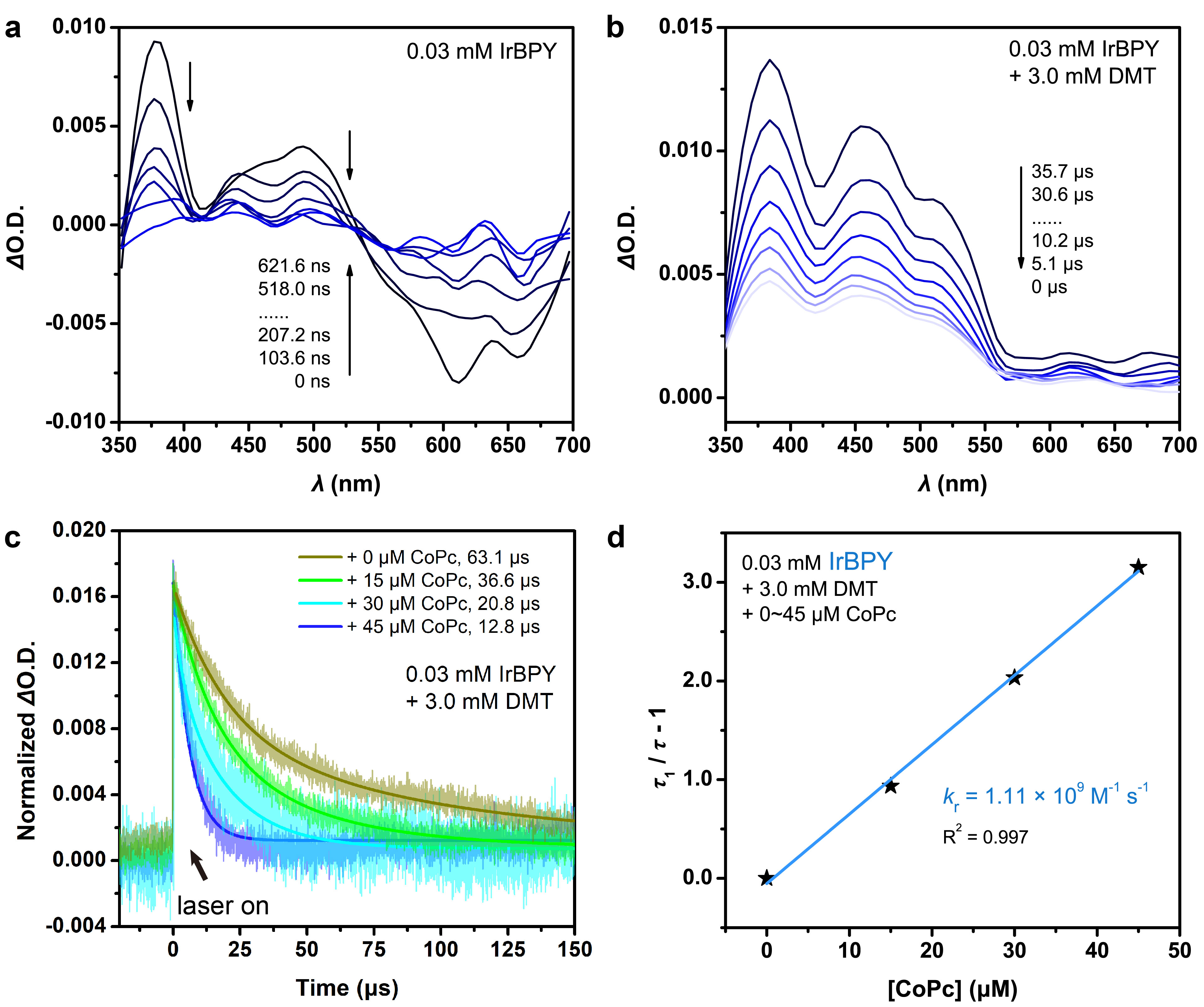


**Supplementary Figure 28** **| Transient absorption spectroscopy**. **a** TA spectrum of 0.03 mM IrBPY. **b** TA spectrum of 0.03 mM IrBPY in the presence of 3.0 mM DMT. **e** TA decay traces of 0.03 mM IrBPY with 3.0 mM DMT in the absence (wheat) or presence of 15 (green), 30 (cyan), 45 (blue) μM CoPc at 350 nm. **f** Plot of (*τ*_1_ / *τ* - 1) versus [CoPc] with linear fitting for the IrBPY system. The excitation wavelength is 355 nm. The temporal evolution profiles of IrBPY* and IrBPY^-^ are similar to those of IrQPY* and IrQPY^-^, respectively, thus a detailed description is omitted.


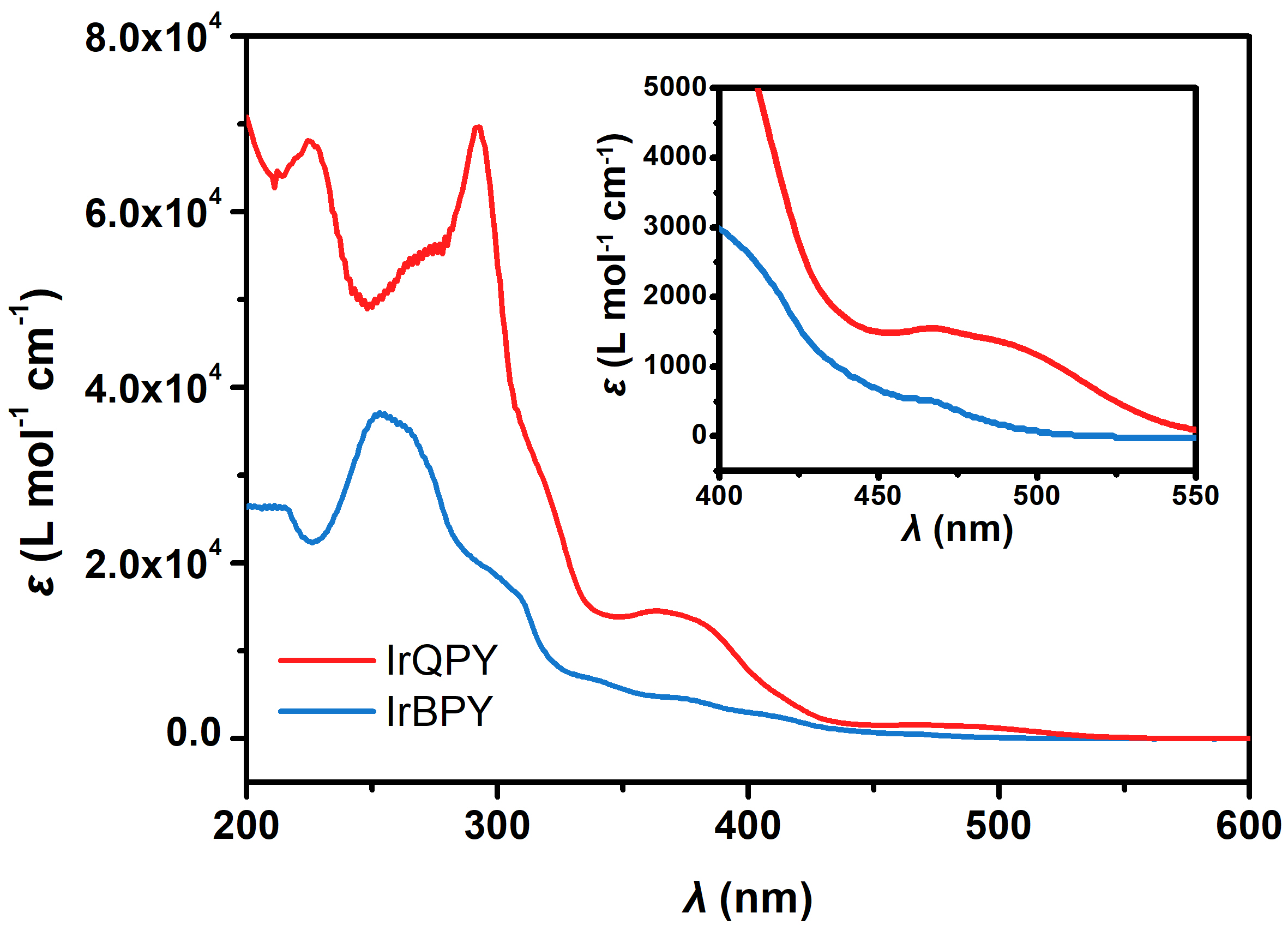


**Supplementary Figure 29 | UV-Vis spectroscopy.** UV-Vis spectra of 50 μM IrQPY (red) and IrBPY (blue) in CH_3_CN.

**Supplementary tables**

**Supplementary Table 1.** Reported QEs from pioneering molecular systems for photocatalytic CO_2_-to-CO conversion with noble-metal PSs and earth-abundant catalysts.

| **Entry** | **Catalyst** | **PS** | **QE (%)** | **CO (%)** | **Ref.** |
| --- | --- | --- | --- | --- | --- |
| 1 |   CoPc |   IrQPY | 10.2±0.5 at 450 nm | 98 | This work |
| 2 |   CoPc |   IrBPY | 2.4±0.2 at 450 nm | 91 | This work |
| 3 |   CoTAPc |   IrQPY | 27.9±0.8 at 425 nm | 99 | This work |
| 4 |  |  | 4.9 at 450 nm | 98 | ^14^ |

| 5 |  |  | 1.43 at 450 nm | 99 | ^15^ |
| --- | --- | --- | --- | --- | --- |
| 6 |  |  | 11.1 at 450 nm | 99 | ^16^ |
| 7 |  |  | 2.8 at 458 nm | 98 | ^17^ |
| 7 |  |  | 8.8 at 458 nm | 95 | ^17^ |

Supplementary Table 2. Chemical shifts of proton signals at the qpy ligand of IrQPY in the ^1^H NMR titration with CoPc. Residuals of the optimal model are shown.

| Host  [IrQPY] (mM) | Guest  [CoPc] (mM) | CoPc/IrQPY (guest/host) equivalents | Proton I* | Proton M | Proton J | Proton K* | Proton L* | Proton I residuals | Proton M residuals | Proton J residuals | Proton K residuals | Proton L residuals |
| --- | --- | --- | --- | --- | --- | --- | --- | --- | --- | --- | --- | --- |
| 0.20 | 0 | 0 | 9.667 | 8.864 | 8.28 | 8.214 | 8.118 | 0.00000 | 0.00000 | 0.00000 | 0.00000 | 0.00000 |
|  | 0.04 | 0.2 | 9.666 | 8.872 | 8.28 | 8.214 | 8.1205 | 0.00024 | -0.00403 | -0.00007 | -0.00009 | -0.00096 |
|  | 0.09 | 0.45 | 9.665 | 8.875 | 8.279 | 8.2135 | 8.1215 | 0.00018 | -0.00279 | 0.00079 | 0.00029 | -0.00043 |
|  | 0.17 | 0.85 | 9.6625 | 8.878 | 8.28 | 8.2135 | 8.124 | 0.00092 | -0.00031 | -0.00051 | 0.00011 | -0.00117 |
|  | 0.25 | 1.25 | 9.6615 | 8.88 | 8.279 | 8.2135 | 8.1225 | 0.00017 | 0.00195 | 0.00014 | -0.00006 | 0.00151 |
|  | 0.33 | 1.65 | 9.661 | 8.884 | 8.279 | 8.2135 | 8.125 | -0.00097 | 0.00133 | -0.00021 | -0.00021 | -0.00019 |
|  | 0.48 | 2.4 | 9.658 | 8.889 | 8.278 | 8.213 | 8.125 | -0.00069 | 0.00103 | 0.00017 | 0.00003 | 0.00070 |
|  | 0.62 | 3.1 | 9.655 | 8.893 | 8.278 | 8.213 | 8.126 | 0.00017 | 0.00016 | -0.00033 | -0.00016 | 0.00012 |
|  | 0.75 | 3.75 | 9.653 | 8.897 | 8.277 | 8.2125 | 8.127 | 0.00049 | -0.00165 | 0.00026 | 0.00019 | -0.00068 |

*The values are the average ones from doublet/quartet proton signals in Supplementary Table 3.

Supplementary Table 3. Doublet/quartet* proton signals of qpy ligand in IrQPY/CoPc ^1^H NMR titration.

| [IrQPY] (mM) | [CoPc] (mM) | Proton I-1 | Proton I-2 | Proton K-1 | Proton K-2 | Proton L-1 | Proton L-2 |
| --- | --- | --- | --- | --- | --- | --- | --- |
| 0.20 | 0 | 9.669 | 9.665 | 8.221 | 8.207 | 8.126 | 8.11 |
|  | 0.04 | 9.668 | 9.664 | 8.221 | 8.207 | 8.128 | 8.113 |
|  | 0.09 | 9.667 | 9.663 | 8.221 | 8.206 | 8.127 | 8.116 |
|  | 0.17 | 9.664 | 9.661 | 8.221 | 8.206 | 8.13 | 8.118 |
|  | 0.25 | 9.663 | 9.66 | 8.221 | 8.206 | 8.126 | 8.119 |
|  | 0.33 | 9.661 | 9.661 | 8.221 | 8.206 | 8.125 | 8.125 |
|  | 0.48 | 9.658 | 9.658 | 8.221 | 8.205 | 8.125 | 8.125 |
|  | 0.62 | 9.655 | 9.655 | 8.221 | 8.205 | 8.126 | 8.126 |
|  | 0.75 | 9.653 | 9.653 | 8.22 | 8.205 | 8.127 | 8.127 |

*One pair of the doublets in the quartets is listed for averaging values.

Supplementary Table 4. Bindfit results for IrQPY/CoPc

| Host:Guest Model | Flavor | Applicable? Y/N | *K* (M^-1^) | *K* error (%) | Total root mean square | Total covariance | Optimal model* |
| --- | --- | --- | --- | --- | --- | --- | --- |
| 1:1 | None | Y | *K*_11_ = 3245.078 | ±9.827 | 0.001173687 | 0.014470088 |  |
| 1:2 | None | N | N.A. | | | |  |
| 1:2 | Non-cooperative | Y | *K*_11_ = *K*_12_ = 4206.404 | ±10.530 | 0.001045778 | 0.001045778 |  |
| 2:1 | None | N | N.A. | | | |  |
| 2:1 | Non-cooperative | Y | *K*_11_ = *K*_21_ = 2361.801 | ±6.739 | 0.00098067 | 0.00098067 | √ |

*It can be seen that the three applicable models all afford the 1:1 binding constants (*K*_11_), which make the related error data comparable. Considering the *K* errors to be within ±10%, the 1:1 and 2:1 non-cooperative models are more reasonable. Finally, with the smaller total root mean square and total covariance, the 2:1 non-cooperative binding mode should be the optimal model to afford the binding constant *K*_11_ = 2362±159 M^-1^. The error bar was calculated according to the corresponding *K* error value. It should be noted that the 2:1 binding of IrQPY/CoPc is possible if two IrQPY molecules both axially coordinate to one CoPc complex from two sides. Notably, here the mathematically optimal model is given, while the 1:1 and 1:2 non-cooperative models should also be reasonable according to the DFT-simulated results (Figure 2).

Supplementary Table 5. Chemical shifts of proton signals at the qpy ligand of IrQPY in the ^1^H NMR titration with ZnPc. Residuals of the optimal model are shown.

| Host  [IrQPY] (mM) | Guest  [ZnPc] (mM) | ZnPc/IrQPY (guest/host) equivalents | Proton I* | Proton M* | Proton J | Proton K* | Proton L* | Proton I residuals | Proton M residuals | Proton J residuals | Proton K residuals | Proton L residuals |
| --- | --- | --- | --- | --- | --- | --- | --- | --- | --- | --- | --- | --- |
| 0.20 | 0 | 0 | 9.667 | 8.864 | 8.28 | 8.214 | 8.118 | 0.00000 | 0.00000 | 0.00000 | 0.00000 | 0.00000 |
|  | 0.04 | 0.2 | 9.6645 | 8.8635 | 8.281 | 8.214 | 8.116 | 0.00007 | 0.00029 | -0.00039 | 0.00006 | 0.00052 |
|  | 0.09 | 0.45 | 9.6625 | 8.8635 | 8.281 | 8.214 | 8.1155 | -0.00072 | 0.00004 | 0.00032 | 0.00011 | -0.00065 |
|  | 0.17 | 0.85 | 9.6575 | 8.863 | 8.283 | 8.214 | 8.1125 | 0.00030 | 0.00017 | -0.00065 | 0.00013 | 0.00002 |
|  | 0.25 | 1.25 | 9.6535 | 8.863 | 8.284 | 8.214 | 8.11 | 0.00082 | -0.00016 | -0.00074 | 0.00009 | 0.00054 |
|  | 0.33 | 1.65 | 9.6505 | 8.8625 | 8.284 | 8.214 | 8.1085 | 0.00077 | 0.00003 | 0.00007 | 0.00001 | 0.00033 |
|  | 0.48 | 2.4 | 9.648 | 8.862 | 8.284 | 8.214 | 8.107 | -0.00153 | 0.00002 | 0.00136 | -0.00018 | -0.00077 |
|  | 0.62 | 3.1 | 9.6435 | 8.862 | 8.286 | 8.214 | 8.1045 | -0.00068 | -0.00038 | 0.00036 | -0.00040 | -0.00018 |
|  | 0.75 | 3.75 | 9.639 | 8.861 | 8.288 | 8.213 | 8.1025 | 0.00097 | 0.00029 | -0.00085 | 0.00038 | 0.00037 |

*The values are the average ones from the doublet/quartet proton signals in Supplementary Table 6.

Supplementary Table 6. Doublet/quartet* proton signals of qpy ligand in IrQPY/ZnPc ^1^H NMR titration.

| [IrQPY] (mM) | [ZnPc] (mM) | Proton I-1 | Proton I-2 | Proton M-1 | Proton M-2 | Proton K-1 | Proton K-2 | Proton L-1 | Proton L-2 |
| --- | --- | --- | --- | --- | --- | --- | --- | --- | --- |
| 0.20 | 0 | 9.669 | 9.665 | 8.8715 | 8.8565 | 8.221 | 8.207 | 8.126 | 8.11 |
|  | 0.04 | 9.666 | 9.663 | 8.8715 | 8.8555 | 8.221 | 8.207 | 8.124 | 8.108 |
|  | 0.09 | 9.664 | 9.661 | 8.8715 | 8.8555 | 8.221 | 8.207 | 8.123 | 8.108 |
|  | 0.17 | 9.659 | 9.656 | 8.8705 | 8.8555 | 8.221 | 8.207 | 8.12 | 8.105 |
|  | 0.25 | 9.655 | 9.652 | 8.8705 | 8.8555 | 8.221 | 8.207 | 8.118 | 8.102 |
|  | 0.33 | 9.653 | 9.648 | 8.8705 | 8.8545 | 8.221 | 8.207 | 8.116 | 8.101 |
|  | 0.48 | 9.65 | 9.646 | 8.8695 | 8.8545 | 8.221 | 8.207 | 8.115 | 8.099 |
|  | 0.62 | 9.644 | 9.643 | 8.8695 | 8.8545 | 8.221 | 8.207 | 8.112 | 8.097 |
|  | 0.75 | 9.641 | 9.637 | 8.8685 | 8.8535 | 8.22 | 8.206 | 8.11 | 8.095 |

*One pair of the doublets in the quartets is listed for averaging values.

Supplementary Table 7. Bindfit results for IrQPY/ZnPc

| Host:Guest Model | Flavor | Applicable? Y/N | *K* (M^-1^) | *K* error (%) | Total root mean square | Total covariance | Optimal model* |
| --- | --- | --- | --- | --- | --- | --- | --- |
| 1:1 | None | Y | *K*_11_ = 1512.962 | ±3.880 | 0.00053688 | 0.00503491 |  |
| 1:2 | None | N | *K*_11_ = 2571.643  *K*_12_ = -845.436 | ±3.656  ±3.655 | 0.00039701 | 0.00274386 |  |
| 1:2 | Non-cooperative | Y | *K*_11_ = *K*_12_ = 2203.823 | ±4.551 | 0.00052841 | 0.00487642 |  |
| 2:1 | None | N | N.A. | | | |  |
| 2:1 | Non-cooperative | Y | *K*_11_ = *K*_21_ = 1064.042 | ±3.140 | 0.00052054 | 0.00473286 | √ |

* The 1:2 binding model is applicable here, while the binding constant for the second molecule, *K*_12_, is a minus value which is not rational. The other three applicable models all afford the 1:1 binding constants (*K*_11_) with *K* errors within ±10%, which make the related error data comparable. With the smallest values of total root mean square and total covariance, the 2:1 non-cooperative binding mode should be the optimal model to afford the binding constant *K*_11_ = 1064±33 M^-1^. The error bar was calculated according to the corresponding *K* error value. Notably, here the mathematically optimal model is given, while the 1:1 and 1:2 non-cooperative models should also be reasonable according to the DFT-simulated results (Figure 2).

Supplementary Table 8. Chemical shifts of proton signals at the qpy ligand of IrQPY in the ^1^H NMR titration with CoPc in the presence of 2.5 v% TEA. Residuals of the optimal model are shown.

| Host  [IrQPY] (mM) | Guest  [CoPc] (mM) | CoPc/IrQPY (guest/host) equivalents | Proton I* | Proton M | Proton J | Proton K* | Proton L* | Proton I residuals | Proton M residuals | Proton J residuals | Proton K residuals | Proton L residuals |
| --- | --- | --- | --- | --- | --- | --- | --- | --- | --- | --- | --- | --- |
| 0.20 | 0 | 0 | 9.667 | 8.864 | 8.28 | 8.214 | 8.118 | 0.00000 | 0.00000 | 0.00000 | 0.00000 | 0.00000 |
|  | 0.04 | 0.2 | 9.666 | 8.867 | 8.28 | 8.214 | 8.121 | 0.00017 | -0.00004 | 0.00033 | 0.00026 | -0.00126 |
|  | 0.09 | 0.45 | 9.665 | 8.872 | 8.281 | 8.214 | 8.1215 | 0.00019 | -0.00150 | -0.00031 | 0.00000 | 0.00024 |
|  | 0.17 | 0.85 | 9.6645 | 8.878 | 8.281 | 8.2135 | 8.1245 | -0.00077 | -0.00214 | 0.00020 | 0.00015 | 0.00007 |
|  | 0.25 | 1.25 | 9.662 | 8.881 | 8.282 | 8.2135 | 8.1265 | 0.00040 | -0.00014 | -0.00037 | -0.00013 | 0.00052 |
|  | 0.33 | 1.65 | 9.661 | 8.885 | 8.282 | 8.213 | 8.1295 | 0.00018 | 0.00053 | -0.00002 | 0.00015 | -0.00036 |
|  | 0.48 | 2.4 | 9.659 | 8.891 | 8.282 | 8.213 | 8.1325 | 0.00016 | 0.00250 | 0.00047 | -0.00012 | -0.00012 |
|  | 0.62 | 3.1 | 9.658 | 8.9 | 8.283 | 8.213 | 8.1345 | -0.00046 | 0.00015 | -0.00021 | -0.00025 | 0.00021 |
|  | 0.75 | 3.75 | 9.656 | 8.907 | 8.283 | 8.2125 | 8.1365 | 0.00021 | -0.00127 | -0.00002 | 0.00021 | -0.00010 |

*The values are the average ones from the doublet/quartet proton signals in Supplementary Table 9.

Supplementary Table 9. Doublet/quartet* proton signals of qpy ligand in IrQPY/CoPc/TEA ^1^H NMR titration.

| [IrQPY] (mM) | [CoPc] (mM) | Proton I-1 | Proton I-2 | Proton K-1 | Proton K-2 | Proton L-1 | Proton L-2 |
| --- | --- | --- | --- | --- | --- | --- | --- |
| 0.20 | 0 | 9.669 | 9.665 | 8.221 | 8.207 | 8.126 | 8.11 |
|  | 0.04 | 9.667 | 9.665 | 8.221 | 8.206 | 8.128 | 8.114 |
|  | 0.09 | 9.667 | 9.663 | 8.221 | 8.206 | 8.128 | 8.115 |
|  | 0.17 | 9.665 | 9.664 | 8.22 | 8.206 | 8.125 | 8.124 |
|  | 0.25 | 9.662 | 9.662 | 8.22 | 8.206 | 8.127 | 8.126 |
|  | 0.33 | 9.661 | 9.661 | 8.22 | 8.205 | 8.13 | 8.129 |
|  | 0.48 | 9.659 | 9.659 | 8.22 | 8.205 | 8.133 | 8.132 |
|  | 0.62 | 9.658 | 9.658 | 8.22 | 8.205 | 8.135 | 8.134 |
|  | 0.75 | 9.656 | 9.656 | 8.219 | 8.205 | 8.137 | 8.136 |

*One pair of the doublets in the quartets is listed for averaging values.

Supplementary Table 10. Binding fitting results for IrQPY/CoPc/TEA system.

| Host:Guest Model | Flavor | Applicable? Y/N | *K* (M^-1^) | *K* error (%) | Total root mean square | Total covariance | Optimal model* |
| --- | --- | --- | --- | --- | --- | --- | --- |
| 1:1 | None | Y | *K*_11_ = 746.356 | ±3.019 | 0.000737933 | 0.00453431 |  |
| 1:2 | None | Y | *K*_11_ = 7717.620  *K*_12_ = -576.128 | ±8.789  ±3.552 | 0.00046901 | 0.00184728 |  |
| 1:2 | Non-cooperative | Y | *K*_11_ = *K*_12_ = 1046.196 | ±3.020 | 0.00065669 | 0.00359917 |  |
| 2:1 | None | N | N.A. | | | |  |
| 2:1 | Non-cooperative | Y | *K*_11_ = *K*_21_ = 516.911 | ±2.020 | 0.00065242 | 0.0035551 | √ |

*The 1:2 binding model is applicable here, while the binding constant for the second molecule, *K*_12_, is a minus value which is not rational. The other three applicable models all afford the 1:1 binding constants (*K*_11_) with *K* errors within ±10%, which make the related error data comparable. With the smallest values of total root mean square and total covariance, the 2:1 non-cooperative binding mode should be the optimal model to afford the binding constant *K*_11_ = 517±10 M^-1^. The error bar was calculated according to the corresponding *K* error value. Notably, here the mathematically optimal model is given, while the 1:1 and 1:2 non-cooperative models should also be reasonable according to the DFT-simulated results (Figure 2).

**Supplementary Table 11.** Photocatalytic CO_2_ reduction to CO by IrQPY/CoPc in the absence of certain component.^*^

| Entry | Conditions | *n*(CO) (*μ*mol) | *n*(H_2_) (*μ*mol) |
| --- | --- | --- | --- |
| 1 | No CoPc | N.D. | 0.08±0.01 |
| 2 | No IrQPY | N.D. | N.D. |
| 3 | Under N_2_ instead of CO_2_ | N.D. | N.D. |
| 4 | No irradiation | N.D. | N.D. |
| 5 | 10% CO_2_/argon | 89.7±2.0 | 18.50±0.55 |
| 6 | No TEA | 66.9±0.8 | 6.50±0.05 |
| 7 | No BIH | N.D. | 0.14±0.02 |

^*^Standard condition: CoPc (0.1 mM), IrQPY (0.1 mM), PhOH (6.0 v%), TEA (2.5 v%), and BIH (80 mM) in 4.0 mL CH_3_CN within 4 h of 450 nm irradiation under 1 atm CO_2_.

**Supplementary Table 12.** Photocatalytic CO_2_ reduction to CO by IrQPY/CoPc with varied volume ratios (v%) of different proton sources.^*^

| Entry | Proton source | Volume ratio (v%) | *n*(CO) (*μ*mol) | *n*(H_2_) (*μ*mol) | CO% |
| --- | --- | --- | --- | --- | --- |
| 1 | No proton source | 0 | 13.9±0.2 | 0.60±0.10 | 96 |
| 2 | PhOH | 2.0 | 106.9±1.4 | 2.30±0.02 | 98 |
| 3 | PhOH | 4.0 | 126.8±0.8 | 2.39±0.14 | 98 |
| 4 | PhOH | 6.0 | 156.4±2.8 | 3.97±0.06 | 98 |
| 5 | PhOH | 8.0 | 145.9±1.9 | 4.71±0.08 | 97 |
| 6 | TFE | 2.0 | 50.1±0.7 | 3.51±0.04 | 93 |
| 7 | TFE | 4.0 | 82.2±3.4 | 11.30±0.04 | 92 |
| 8 | TFE | 6.0 | 62.4±0.5 | 2.30±0.02 | 85 |
| 9 | H_2_O | 2.0 | 34.2±1.2 | 2.48±0.10 | 93 |
| 10 | H_2_O | 4.0 | 56.3±1.0 | 3.74±0.08 | 94 |
| 11 | H_2_O | 6.0 | 66.9±2.0 | 4.98±0.07 | 93 |
| 12 | H_2_O | 10.0 | 34.8±0.3 | 2.21±0.03 | 94 |
| 13 | H_2_O | 25.0 | 24.1±0.2 | 1.67±0.10 | 93 |
| 14 | H_2_O | 50.0 | 1.1±0.1 | 12.15±0.90 | 8.3 |

^*^Condition: CoPc (0.1 mM), IrQPY (0.1 mM), TEA (2.5 v%), and BIH (80 mM) in 4.0 mL CH_3_CN within 4 h of 450 nm irradiation under 1 atm CO_2_.

**Supplementary Table 13.** Photocatalytic CO_2_ reduction to CO for QE determination.^*^

| Entry | Catalysts | Ir PSs | BIH (mM) | Time (h) | Wavelength (nm) | n(CO) (μmol) | n(H_2_) (μmol) | QE(CO)  (%) |
| --- | --- | --- | --- | --- | --- | --- | --- | --- |
| 1 | CoPc | IrQPY | 80 | 1.0 | 450 | 110.2±5.5 | 3.09±0.26 | 20.4±1.0 |
| 2 | CoPc | IrBPY | 80 | 1.0 | 450 | 26.5±2.4 | 1.39±0.17 | 4.9±0.4 |
| 3 | CoPc | IrQPY | 150 | 0.5 | 450 | 78.5±4.2 | 1.59±0.07 | 29.1±2.6 |
| 4 | CoPc | IrQPY | 150 | 0.5 | 425 | 109.9±2.3 | 1.82±0.14 | 38.4±0.8 |
| 5 | CoPc | IrQPY | 150 | 0.5 | 405 | 99.9±3.4 | 1.93±0.08 | 33.3±1.1 |
| 6 | CoPc | IrBPY | 150 | 0.5 | 425 | 74.4±9.6 | 2.06±0.20 | 26.0±3.4 |
| 7 | CoTAPc | IrQPY | 150 | 0.5 | 425 | 159.4±4.7 | 2.15±0.07 | 55.7±1.6 |
| 8 | CoTAPc | IrBPY | 150 | 0.5 | 425 | 56.2±6.3 | 1.81±0.25 | 18.7±2.1 |

^*^Standard condition: Cobalt phthalocyanine catalysts (0.1 mM), Ir PSs (0.1 mM), PhOH (6.0 v%), TEA (2.5 v%), and BIH (80 or 150 mM) in 4.0 mL CH_3_CN within LED irradiation under 1 atm CO_2_.

**Supplementary Table 14.** Emission quantum yields of IrQPY.

| Entry | Excitation *λ* (nm) | *Φ* (%) |
| --- | --- | --- |
| 1 | 450 | 6.86 |
| 2 | 425 | 11.84 |
| 3 | 405 | 7.72 |

Supplementary Table 15. Chemical shifts of proton signals at the qpy ligand of IrQPY in the ^1^H NMR titration with CoTAPc. Residuals of the optimal model are shown.

| Host  [IrQPY] (mM) | Guest  [CoTAPc] (mM) | CoTAPc/IrQPY  (guest/host)  equivalents | Proton I* | Proton M^#^ | Proton J* | Proton K* | Proton L^#^ | Proton I residuals | Proton J residuals | Proton K residuals |
| --- | --- | --- | --- | --- | --- | --- | --- | --- | --- | --- |
| 0.20 | 0 | 0 | 9.667 | 8.864 | 8.28 | 8.214 | 8.118 | 0.00000 | 0.00000 | 0.00000 |
|  | 0.04 | 0.2 | 9.6625 | 8.9055 | 8.279 | 8.211 | 8.146 | 0.00005 | -0.00096 | 0.00039 |
|  | 0.09 | 0.45 | 9.657 | N.A. | 8.276 | 8.207 | N.A. | -0.00100 | -0.00022 | 0.00038 |
|  | 0.17 | 0.85 | 9.644 | N.A. | 8.272 | 8.2 | N.A. | 0.00074 | 0.00062 | 0.00028 |
|  | 0.25 | 1.25 | 9.635 | N.A. | 8.27 | 8.193 | N.A. | -0.00121 | -0.00002 | 0.00026 |
|  | 0.33 | 1.65 | 9.623 | N.A. | 8.267 | 8.1865 | N.A. | 0.00078 | 0.00079 | 0.00028 |
|  | 0.48 | 2.4 | 9.607 | N.A. | 8.2655 | 8.1765 | N.A. | 0.00089 | -0.00092 | -0.00207 |
|  | 0.62 | 3.1 | 9.594 | N.A. | 8.261 | 8.166 | N.A. | 0.00011 | -0.00064 | -0.00029 |
|  | 0.75 | 3.75 | 9.582 | N.A. | 8.2555 | 8.158 | N.A. | -0.00075 | 0.00075 | 0.00138 |

*The values are the average ones from the doublet/quartet proton signals in Supplementary Table 16.

^#^The signals are invisible during titration, which can be attributed to the conditions that the exchange rates between the specific protons and guests are similar to the relaxation time of the NMR machine.

Supplementary Table 16. Doublet/quartet* proton signals in IrQPY/CoTAPc ^1^H NMR titration.

| [IrQPY] (mM) | [CoTAPc] (mM) | Proton I-1 | Proton I-2 | Proton J-1 | Proton J-2 | Proton K-1 | Proton K-2 |
| --- | --- | --- | --- | --- | --- | --- | --- |
| 0.20 | 0 | 9.669 | 9.665 | 8.2825 | 8.2775 | 8.221 | 8.207 |
|  | 0.04 | 9.664 | 9.661 | 8.2825 | 8.2755 | 8.218 | 8.204 |
|  | 0.09 | 9.657 | 9.657 | 8.2825 | 8.2695 | 8.214 | 8.2 |
|  | 0.17 | 9.644 | 9.644 | 8.2815 | 8.2625 | 8.207 | 8.193 |
|  | 0.25 | 9.635 | 9.635 | 8.2815 | 8.2585 | 8.2 | 8.186 |
|  | 0.33 | 9.623 | 9.623 | 8.2815 | 8.2525 | 8.193 | 8.18 |
|  | 0.48 | 9.607 | 9.607 | 8.2825 | 8.2485 | 8.184 | 8.169 |
|  | 0.62 | 9.594 | 9.594 | 8.2825 | 8.2395 | 8.172 | 8.16 |
|  | 0.75 | 9.582 | 9.582 | 8.2825 | 8.2285 | 8.158 | 8.158 |

*One pair of the doublets in the quartets is listed for averaging values.

Supplementary Table 17. Binding fitting results for IrQPY/CoTAPc system.

| Host:Guest Model | Flavor | Applicable? Y/N | *K* (M^-1^) | *K* error (%) | Total root mean square | Total covariance | Optimal model* |
| --- | --- | --- | --- | --- | --- | --- | --- |
| 1:1 | None | Y | *K*_11_ = 647.574 | ±2.498 | 0.00126238 | 0.00324469 |  |
| 1:2 | None | Y | *K*_11_ = -376.668  *K*_12_ = 357.288 | ±0.993  ±0.993 | 0.00117269 | 0.00264779 |  |
| 1:2 | Non-cooperative | Y | *K*_11_ = *K*_12_ = 5244.097 | ±3.670 | 0.00126134 | 0.00306419 |  |
| 2:1 | None | Y | *K*_11_ = 3282.759  *K*_21_ = 2014.349 | ±8.974  ±11.198 | 0.00075795 | 0.00117876 |  |
| 2:1 | Non-cooperative | Y | *K*_11_ = *K*_21_ = 3426.383 | ±6.425 | 0.00075988 | 0.00118455 | √ |

*The 1:2 binding model is applicable here, while the binding constant for the first molecule, *K*_11_, is a minus value which is not rational. The 2:1 binding model is also applicable, while the *K* error exceeds ±10%. The other three applicable models all afford the 1:1 binding constants (*K*_11_) with *K* error within ±10%, which make the related error data comparable. With the smallest values of total root mean square and total covariance, the 2:1 non-cooperative binding mode should be the optimal model to afford the binding constant *K*_11_ = 3426±220 M^-1^. The error bar was calculated according to the corresponding *K* error value. Notably, here the mathematically optimal model is given, while the 1:1 and 1:2 non-cooperative models should also be reasonable according to the DFT-simulated results (Figure 2).

Supplementary Table 18. *ε* values for IrQPY, CoPc and BIH at varying wavelengths.

| Component | *ε*_450_ (M^-1^ cm^-2^) | *ε*_425_ (M^-1^ cm^-2^) | *ε*_405_ (M^-1^ cm^-2^) |
| --- | --- | --- | --- |
| IrQPY | 1500 | 2800 | 6500 |
| CoPc | 8900 | 10000 | 1100 |
| BIH | <10 | <10 | <10 |

**Reference:**

1. DiSalle, B. F. & Bernhard, S. Orchestrated photocatalytic water reduction using surface-adsorbing iridium photosensitizers. *J. Am. Chem. Soc.* **133**, 11819-11821 (2011).

2. Bindfit. <http://app.supramolecular.org/bindfit/> v0.5 edn.

3. Thordarson, P. Determining association constants from titration experiments in supramolecular chemistry. *Chem. Soc. Rev.* **40**, 1305-1323 (2011).

4. Frisch, M. J.*, et al.* *Gaussian 09*, *Revision C.01* (Gaussian, Inc., 2009)

5. Zhao, Y. & Truhlar, D. G. The m06 suite of density functionals for main group thermochemistry, thermochemical kinetics, noncovalent interactions, excited states, and transition elements: Two new functionals and systematic testing of four m06-class functionals and 12 other functionals. *Theor. Chem. Acc.* **120**, 215-241 (2007).

6. Hay, P. J. & Wadt, W. R. Ab initio effective core potentials for molecular calculations. Potentials for k to au including the outermost core orbitals. *J. Chem. Phys.* **82**, 299-310 (1985).

7. Figgis, B. N., Kucharski, E. S. & Reynolds, P. A. Electronic structure of cobalt phthalocyanine: A charge density study. *J. Am. Chem. Soc.* **111**, 1683-1692 (1989).

8. Lu, T. & Chen, F. Multiwfn: A multifunctional wavefunction analyzer. *J. Comput. Chem.* **33**, 580-592 (2012).

9. Guo, Z.*, et al.* Selectivity control of CO versus HCOO^−^ production in the visible-light-driven catalytic reduction of CO_2_ with two cooperative metal sites. *Nat. Catal.* **2**, 801-808 (2019).

10. Kuhn, H., Braslavsky, S. & Schmidt, R. Chemical actinometry (IUPAC technical report). *Pure Appl. Chem.* **76**, 2105-2146 (2004).

11. Thoi, V. S., Kornienko, N., Margarit, C. G., Yang, P. & Chang, C. J. Visible-light photoredox catalysis: Selective reduction of carbon dioxide to carbon monoxide by a nickel *N*-heterocyclic carbene-isoquinoline complex. *J. Am. Chem. Soc.* **135**, 14413-14424 (2013).

12. Wang, P.*, et al.* A broadband and strong visible-light-absorbing photosensitizer boosts hydrogen evolution. *Nat. Commun.* **10**, 3155 (2019).

13. Bhattacharyya, A., Mukherjee, S., Chadha, A. & Prasad, E. Diffusion of solvent-separated ion pairs controls back electron transfer rate in graphene quantum dots. *J. Phys. Chem. C* **122**, 15819-15825 (2018).

14. Ouyang, T.*, et al.* Dinuclear metal synergistic catalysis boosts photochemical CO_2_-to-CO conversion. *Angew. Chem. Int. Ed.* **57**, 16480-16485 (2018).

15. Hong, D., Tsukakoshi, Y., Kotani, H., Ishizuka, T. & Kojima, T. Visible-light-driven photocatalytic CO_2_ reduction by a Ni(II) complex bearing a bioinspired tetradentate ligand for selective CO production. *J. Am. Chem. Soc.* **139**, 6538-6541 (2017).

16. Hong, D., Kawanishi, T., Tsukakoshi, Y., Kotani, H., Ishizuka, T. & Kojima, T. Efficient photocatalytic CO_2_ reduction by a Ni(II) complex having pyridine pendants through capturing a Mg(2+) ion as a lewis-acid cocatalyst. *J. Am. Chem. Soc.* **141**, 20309-20317 (2019).

17. Guo, Z.*, et al.* Highly efficient and selective photocatalytic CO_2_ reduction by iron and cobalt quaterpyridine complexes. *J. Am. Chem. Soc.* **138**, 9413-9416 (2016).

**Appendix: Optimized Coordinates (xyz)** **at the M06/BSI Level**

CoPc low-spin

Co 0.00000000 0.00000000 0.00005700

N -1.35053300 1.36981200 -0.00004100

C -2.70858400 1.17033900 -0.00005100

C -3.39381200 2.44706000 0.00001400

C -4.74347900 2.78421700 0.00003900

H -5.50995500 2.01177900 0.00001600

C -5.06331200 4.13574100 0.00006600

H -6.10836700 4.44117700 0.00006700

C -4.06273000 5.12191200 0.00009000

H -4.35303100 6.17127300 0.00010800

C -2.71607300 4.78244600 0.00008400

H -1.93259000 5.53762600 0.00009800

C -2.39860400 3.42807000 0.00003600

C -1.13172700 2.72485100 -0.00003600

N 0.02380800 3.35680900 -0.00004600

N 1.36974800 1.35059800 -0.00012100

C 1.17032900 2.70865000 -0.00009700

C 2.44707300 3.39383500 -0.00009100

C 2.78428700 4.74348800 -0.00006200

H 2.01188400 5.51000000 -0.00000100

C 4.13582500 5.06326500 -0.00013300

H 4.44129900 6.10830900 -0.00013000

C 5.12195700 4.06264500 -0.00021600

H 6.17132900 4.35290800 -0.00027500

C 4.78243600 2.71600300 -0.00022700

H 5.53758000 1.93248600 -0.00029800

C 3.42804700 2.39859100 -0.00017100

C 2.72478500 1.13173800 -0.00021100

N 3.35671200 -0.02380700 -0.00025500

N 1.35053300 -1.36981200 -0.00005700

C 2.70858400 -1.17033900 -0.00018700

C 3.39381200 -2.44706000 -0.00014300

C 4.74347900 -2.78421700 -0.00021400

H 5.50995500 -2.01177900 -0.00031900

C 5.06331100 -4.13574100 -0.00017800

H 6.10836700 -4.44117700 -0.00024900

C 4.06272900 -5.12191200 -0.00005300

H 4.35303100 -6.17127300 -0.00002900

C 2.71607300 -4.78244600 0.00003400

H 1.93259000 -5.53762600 0.00013100

C 2.39860400 -3.42807000 -0.00002100

C 1.13172700 -2.72485100 -0.00000600

N -0.02380800 -3.35680900 0.00009300

N -1.36974800 -1.35059800 0.00002500

C -1.17032900 -2.70865000 0.00010400

C -2.44707300 -3.39383500 0.00024100

C -2.78428700 -4.74348800 0.00036300

H -2.01188400 -5.51000000 0.00040800

C -4.13582500 -5.06326500 0.00040200

H -4.44129900 -6.10830900 0.00047600

C -5.12195700 -4.06264600 0.00033800

H -6.17132900 -4.35290800 0.00036500

C -4.78243600 -2.71600400 0.00023700

H -5.53758000 -1.93248600 0.00017900

C -3.42804700 -2.39859100 0.00017900

C -2.72478500 -1.13173800 0.00002400

N -3.35671200 0.02380700 -0.00003000

CoPc low-spin

Co 0.00000000 0.00000000 -0.00000900

N 1.36305600 -1.35784500 -0.00001700

C 1.14419600 -2.73786300 -0.00007200

C 2.41598600 -3.42753100 -0.00007500

C 2.75447000 -4.77538500 -0.00011800

H 1.98377900 -5.54366100 -0.00016300

C 4.10958500 -5.09564700 -0.00010400

H 4.41252600 -6.14151100 -0.00013600

C 5.09541600 -4.09977500 -0.00004800

H 6.14484600 -4.38898100 -0.00003900

C 4.75107500 -2.74945700 -0.00000600

H 5.50335100 -1.96311000 0.00003800

C 3.40083600 -2.43602600 -0.00002000

C 2.70421000 -1.15600100 0.00001200

N 3.37338800 -0.01043400 0.00006500

N 1.37146600 1.34934400 0.00008100

C 2.71133800 1.13893300 0.00009800

C 3.41604000 2.41473000 0.00015800

C 4.76825900 2.71905600 0.00018500

H 5.51512500 1.92755100 0.00016300

C 5.12158300 4.06705400 0.00023900

H 6.17286800 4.34944100 0.00026000

C 4.14232600 5.06932800 0.00026600

H 4.45204500 6.11320500 0.00030700

C 2.78504100 4.75802800 0.00023900

H 2.01942300 5.53137700 0.00026000

C 2.43765100 3.41249300 0.00018400

C 1.16136300 2.73078000 0.00013800

N 0.01070300 3.36649700 0.00014700

N -1.36305600 1.35784500 0.00000900

C -1.14419700 2.73786300 0.00009300

C -2.41598600 3.42753100 0.00010300

C -2.75447000 4.77538500 0.00016300

H -1.98377900 5.54366100 0.00022500

C -4.10958500 5.09564700 0.00014400

H -4.41252600 6.14151100 0.00018900

C -5.09541600 4.09977500 0.00006600

H -6.14484600 4.38898100 0.00005300

C -4.75107500 2.74945700 0.00000600

H -5.50335100 1.96311000 -0.00005600

C -3.40083600 2.43602600 0.00002700

C -2.70421000 1.15600100 -0.00002000

N -3.37338800 0.01043400 -0.00009200

N -1.37146600 -1.34934400 -0.00008700

C -2.71133800 -1.13893300 -0.00012200

C -3.41604000 -2.41473000 -0.00018200

C -4.76825900 -2.71905600 -0.00022100

H -5.51512500 -1.92755100 -0.00021600

C -5.12158300 -4.06705400 -0.00026500

H -6.17286800 -4.34944100 -0.00029400

C -4.14232600 -5.06932800 -0.00027000

H -4.45204400 -6.11320500 -0.00030300

C -2.78504100 -4.75802800 -0.00023200

H -2.01942200 -5.53137700 -0.00023400

C -2.43765100 -3.41249300 -0.00018700

C -1.16136300 -2.73078000 -0.00013000

N -0.01070300 -3.36649700 -0.00012100

IrQPY

C -1.62362500 0.72717000 0.15410200

C -2.78817400 1.48506600 0.21493100

H -3.75740900 1.01972000 0.05078100

C -2.72689100 2.86270600 0.43928200

C -1.45968600 3.43150300 0.59577000

H -1.34647900 4.49478500 0.79499100

C -0.33782000 2.62397600 0.52086000

H 0.66758300 3.02944400 0.63835400

C -3.95137200 3.67979100 0.49866100

C -5.13150700 3.17625800 1.05013500

H -5.16878800 2.17758500 1.48298300

C -6.25835100 3.98980400 1.07923700

H -7.18863900 3.61930200 1.51235000

C -5.15709500 5.71005700 0.07686700

H -5.19904900 6.72806800 -0.31260400

C -3.97269500 4.98522200 0.00324600

H -3.09151800 5.42403300 -0.46236200

C -1.62326100 -0.73167500 -0.09148200

C -2.78767000 -1.48880400 -0.16688100

H -3.76089500 -1.01691500 -0.05250500

C -2.72528000 -2.86178900 -0.41683900

C -1.45756100 -3.42891900 -0.57514800

H -1.34214300 -4.49703700 -0.74515200

C -0.33580900 -2.62362500 -0.47910100

H 0.67080000 -3.02908900 -0.58598500

C -3.94961700 -3.67481500 -0.51734300

C -5.06386900 -3.41752000 0.28413500

H -5.04512300 -2.63132200 1.03765400

C -6.19371200 -4.21453200 0.13545300

H -7.07207600 -4.03687300 0.75748600

C -5.21806900 -5.45654100 -1.50216700

H -5.31220600 -6.27529900 -2.21681500

C -4.03574800 -4.72834000 -1.42992100

H -3.20833700 -4.96093900 -2.09854700

C 2.37511200 1.71325800 -2.12319300

C 2.54463500 2.24203600 -3.40604200

H 3.27911200 3.02665800 -3.56810300

C 1.79237100 1.75976700 -4.46177600

H 1.92912200 2.16859300 -5.46068800

C 0.86365300 0.74452500 -4.23447800

H 0.25725400 0.33515100 -5.03732300

C 0.73092500 0.25677200 -2.94903100

H 0.03345900 -0.54406000 -2.70859700

C 3.10915700 2.10823100 -0.93047700

C 2.79723900 1.37882100 0.24226100

C 3.48278500 1.70897800 1.41488400

H 3.28412000 1.16068500 2.33705400

C 4.43120200 2.72810400 1.42667700

H 4.95489300 2.96614700 2.35169100

C 4.72345300 3.44399600 0.26545900

H 5.46694800 4.23778400 0.28195000

C 4.06321900 3.13303100 -0.91226500

H 4.29644400 3.69244000 -1.81823600

C 2.41639200 -1.71665300 2.13366600

C 2.61284500 -2.24572300 3.41244300

H 3.34705000 -3.03390900 3.55814300

C 1.88793300 -1.75865500 4.48502100

H 2.04527500 -2.16762300 5.48082500

C 0.95987000 -0.73834600 4.27854800

H 0.37577700 -0.32408800 5.09531600

C 0.79933600 -0.25110100 2.99603800

H 0.10087800 0.55321300 2.77083900

C 3.12102900 -2.11527500 0.92452100

C 2.78772400 -1.38290800 -0.24026900

C 3.44282900 -1.71741700 -1.42889200

H 3.22598300 -1.16703500 -2.34573600

C 4.38280400 -2.74383000 -1.46328900

H 4.88277200 -2.98527500 -2.40047600

C 4.69641600 -3.46267600 -0.30951400

H 5.43289500 -4.26241600 -0.34394500

C 4.06637000 -3.14733100 0.88357500

H 4.31529700 -3.70955300 1.78360600

Ir 1.35274300 -0.00010100 0.01678100

N -0.40988200 1.30415400 0.30723900

N -6.28461200 5.23337900 0.60252500

N -0.40928500 -1.30677800 -0.24829400

N -6.28352100 -5.21424900 -0.74017300

N 1.46072300 0.72297800 -1.92209000

N 1.50212700 -0.72234100 1.95291900

IrQPY-CoPc

C -2.70286000 0.49851100 -0.12823700

C -1.31529300 0.59598500 -0.14879100

H -0.83299800 1.56174100 -0.27823800

C -0.52210800 -0.54839600 -0.03892600

C -1.17809200 -1.77791900 0.07127400

H -0.61760200 -2.70376400 0.17888300

C -2.56193200 -1.80949600 0.08178400

H -3.11099500 -2.74663200 0.17805500

C 0.94843800 -0.45572300 -0.02985800

C 1.59736000 0.62597400 0.57012600

H 1.03704000 1.41507000 1.06836500

C 2.98423900 0.66696500 0.56777900

H 3.51900200 1.49499300 1.03413800

C 3.12220800 -1.32018100 -0.56295200

H 3.76780000 -2.07615500 -1.01009700

C 1.74076300 -1.44912000 -0.60890800

H 1.29466700 -2.30251000 -1.11615600

C -3.59911200 1.67134300 -0.23181800

C -3.13264400 2.97956000 -0.30968000

H -2.06497500 3.18499500 -0.31674100

C -4.02813900 4.04713100 -0.40891200

C -5.39200000 3.74088700 -0.42459300

H -6.14043200 4.52838100 -0.47418300

C -5.79357500 2.41904500 -0.34202000

H -6.84766900 2.14023600 -0.34449800

C -3.55153000 5.43869600 -0.49595700

C -2.41406900 5.86228200 0.19448700

H -1.86406600 5.18366800 0.84481200

C -2.01269600 7.18829300 0.07528100

H -1.13254700 7.54212100 0.61395800

C -3.73410500 7.68110800 -1.32851100

H -4.24018200 8.43135300 -1.93741900

C -4.22592200 6.38179700 -1.27443300

H -5.10349300 6.10689100 -1.85746200

C -5.66258300 -2.58592100 -2.24008100

C -5.70350100 -3.04879000 -3.55827500

H -5.87010100 -4.10620100 -3.74734500

C -5.54697100 -2.16478500 -4.61013000

H -5.58488500 -2.52411100 -5.63624200

C -5.34366400 -0.81116100 -4.34234000

H -5.21648700 -0.08379700 -5.13903600

C -5.31203800 -0.40302200 -3.02297800

H -5.16720500 0.64105000 -2.75024000

C -5.82317500 -3.40027500 -1.04528600

C -5.81613300 -2.68832800 0.17834700

C -5.96374800 -3.42354900 1.35807000

H -5.97943100 -2.91441700 2.32291000

C -6.10043200 -4.80880100 1.32628100

H -6.21378100 -5.35955600 2.25921100

C -6.09888700 -5.49818900 0.11351000

H -6.20740600 -6.58031600 0.09585200

C -5.96260800 -4.79301800 -1.07132800

H -5.96227900 -5.33375700 -2.01780800

C -7.05502400 -0.11267200 2.41625400

C -7.33111800 0.10551100 3.76923300

H -8.35867100 0.26616400 4.08518700

C -6.30565000 0.10444100 4.69725900

H -6.52222900 0.26847800 5.75071300

C -4.99582400 -0.11284800 4.27023400

H -4.16264700 -0.12624900 4.96697800

C -4.77422900 -0.32155200 2.92328000

H -3.77622800 -0.51041900 2.53124100

C -8.03259700 -0.15719400 1.33976700

C -7.51276900 -0.46149100 0.05815700

C -8.41237800 -0.51882500 -1.01041500

H -8.05685000 -0.76443600 -2.01231400

C -9.77010700 -0.27705500 -0.81723000

H -10.45220900 -0.32940900 -1.66487900

C -10.26753000 0.02435300 0.45055200

H -11.32991900 0.20833600 0.59470800

C -9.39864900 0.08339500 1.52813600

H -9.79097700 0.31877600 2.51739200

Ir -5.51789300 -0.70908200 -0.00159300

N -3.31103800 -0.70335600 -0.00540100

N 3.73703900 -0.28566900 0.01437200

N -4.92508800 1.40387200 -0.25059500

N -2.65003400 8.08956600 -0.67048700

N -5.46921300 -1.25876600 -1.99921100

N -5.76848800 -0.32301700 2.01974800

Co 5.99569400 -0.15183600 0.06495300

N 6.17075800 -0.77393500 -1.75482700

C 6.14072600 0.00898800 -2.87929200

C 6.25888600 -0.82288700 -4.06185500

C 6.28815900 -0.52257100 -5.41961500

H 6.21329500 0.50734200 -5.76349700

C 6.42202800 -1.58374600 -6.30636100

H 6.45153300 -1.38949700 -7.37717400

C 6.52772300 -2.90707400 -5.84839900

H 6.63779800 -3.71102500 -6.57422100

C 6.50244100 -3.20430700 -4.49157400

H 6.59221400 -4.22497600 -4.12426200

C 6.36532700 -2.14030400 -3.60631200

C 6.30567200 -2.07664600 -2.15834800

N 6.36451000 -3.15637800 -1.40086400

N 6.19054700 -1.96804600 0.69438200

C 6.32330500 -3.08832400 -0.08333200

C 6.40621900 -4.26694300 0.75881400

C 6.55372800 -5.61917600 0.46846000

H 6.63077000 -5.96228400 -0.56157800

C 6.60565800 -6.50113600 1.54095500

H 6.72484700 -7.56730900 1.35555100

C 6.51486600 -6.04370800 2.86551000

H 6.56573100 -6.76493000 3.67943600

C 6.36919500 -4.69235400 3.15284900

H 6.30497900 -4.32523900 4.17541500

C 6.31440400 -3.81227900 2.07765000

C 6.18189100 -2.36946800 2.00426300

N 6.08512700 -1.61656700 3.08212200

N 6.00532500 0.48227300 1.88993300

C 6.02134900 -0.30108500 3.01330400

C 5.97118100 0.53480900 4.19792100

C 5.97033100 0.23524400 5.55625700

H 6.00907300 -0.79744800 5.89774100

C 5.92380200 1.30124500 6.44603300

H 5.92462600 1.10820400 7.51749000

C 5.88269500 2.62900500 5.99038000

H 5.85292200 3.43807600 6.71829900

C 5.88606600 2.92594000 4.63325900

H 5.86276300 3.95151800 4.26933600

C 5.92982800 1.85671800 3.74447200

C 5.94919400 1.79044500 2.29555200

N 5.89937800 2.87045900 1.53801400

N 5.98503600 1.67654600 -0.56021100

C 5.92887300 2.80153900 0.21995100

C 5.88651700 3.98295000 -0.62037100

C 5.83582100 5.34156800 -0.32571800

H 5.82461100 5.68660800 0.70658000

C 5.81090400 6.22719300 -1.39589300

H 5.77610700 7.29888700 -1.20709800

C 5.83837700 5.76713700 -2.72250500

H 5.82294000 6.49183100 -3.53482900

C 5.89212800 4.40984900 -3.01408300

H 5.92162700 4.04264600 -4.03824000

C 5.91412000 3.52501400 -1.94114300

C 5.97863100 2.07731900 -1.86985400

N 6.03324700 1.32107600 -2.94904500

IrQPY-(CoPc)_2_

C 0.70652000 3.26637200 -0.19257500

C 1.38371400 2.05282200 -0.12844100

H 0.83400800 1.11544100 -0.09061100

C 2.77827500 2.02302200 -0.06970000

C 3.45004800 3.24886700 -0.07404300

H 4.53668600 3.29017700 -0.05336300

C 2.71878500 4.42287700 -0.13660200

H 3.20448800 5.39917100 -0.15041200

C 3.50974400 0.74518500 -0.00729700

C 3.05921200 -0.37964300 -0.70144200

H 2.16949700 -0.33894500 -1.32712000

C 3.79263700 -1.55539800 -0.62758300

H 3.48072300 -2.44690000 -1.17351900

C 5.34150200 -0.59878000 0.76404900

H 6.25373800 -0.73000800 1.34718000

C 4.68065000 0.62175200 0.74349900

H 5.06457700 1.45570800 1.32819500

C -0.76812800 3.36454100 -0.25376800

C -1.60146200 2.25286900 -0.30830200

H -1.18692500 1.24807500 -0.29000800

C -2.98841500 2.40942500 -0.34714600

C -3.49255300 3.71244100 -0.33768600

H -4.56340500 3.89665800 -0.38856300

C -2.61026900 4.77846500 -0.28959000

H -2.95970300 5.81125400 -0.28445200

C -3.87102300 1.23075500 -0.37174600

C -3.52078300 0.08322900 -1.08626800

H -2.62336400 0.05339500 -1.70163900

C -4.34828300 -1.02783300 -1.02163700

H -4.10161300 -1.94041000 -1.56510500

C -5.82455400 0.04850300 0.35937200

H -6.74696000 -0.01178400 0.93852200

C -5.06759800 1.21258000 0.34700600

H -5.38564600 2.07259500 0.93374400

C 1.37532300 7.21032100 2.29921400

C 1.58478200 7.36829000 3.67219500

H 2.34748700 8.05983200 4.02085800

C 0.81617900 6.66158200 4.57895000

H 0.97518900 6.79053400 5.64741600

C -0.16582700 5.78896400 4.11047700

H -0.79303200 5.21718500 4.78839400

C -0.33349100 5.66640400 2.74489700

H -1.09234200 5.01207100 2.31879400

C 2.09384900 7.90343300 1.24095400

C 1.64873400 7.63031900 -0.07496900

C 2.30409800 8.27436000 -1.12849500

H 1.98516200 8.10507100 -2.15805600

C 3.36436200 9.14430600 -0.88755200

H 3.85923600 9.63526000 -1.72463800

C 3.79650500 9.39894800 0.41415600

H 4.62366600 10.08177000 0.59501200

C 3.16007500 8.77938400 1.47728300

H 3.49961700 8.98023800 2.49329800

C -0.93162900 7.44813300 -2.64430900

C -1.12738500 7.68052000 -4.00888700

H -1.80940300 8.46562000 -4.32541500

C -0.44660800 6.92644400 -4.94706200

H -0.59450900 7.11186900 -6.00880500

C 0.43420400 5.93348600 -4.51858200

H 0.99410300 5.32413200 -5.22220200

C 0.59032000 5.73977200 -3.15981900

H 1.27453300 4.99126500 -2.76336500

C -1.56097400 8.17960400 -1.55516500

C -1.14872800 7.80374600 -0.25395700

C -1.72347900 8.47579100 0.82884600

H -1.42754500 8.22622000 1.84910200

C -2.67403800 9.47360200 0.62915100

H -3.10823700 9.98329600 1.48837900

C -3.07365700 9.83104000 -0.65861900

H -3.81591900 10.61229700 -0.80674600

C -2.51622000 9.18440700 -1.74973200

H -2.83085500 9.46471800 -2.75483400

Ir 0.16203400 6.28124200 -0.19551600

N 1.38117200 4.43898200 -0.19820100

N 4.91430400 -1.66720200 0.08699900

N -1.28181000 4.61563000 -0.24237500

N 0.41063600 6.35289700 1.86171700

N -0.07081700 6.46973500 -2.24619700

Co 6.18850200 -3.54475200 -0.06617100

N 6.91346200 -3.34354200 1.71146200

C 6.37939500 -3.86372600 2.86137400

C 7.18000600 -3.45997700 4.00207400

C 7.06655300 -3.72022900 5.36345200

H 6.25252600 -4.33292000 5.74623200

C 8.03024500 -3.17299500 6.20129000

H 7.97739300 -3.35497600 7.27330300

C 9.07946200 -2.39104100 5.69142400

H 9.81836900 -1.98315600 6.37912200

C 9.19291800 -2.13583900 4.33046600

H 10.00647400 -1.53765600 3.92443500

C 8.22523100 -2.68262000 3.49422600

C 8.03079700 -2.62806200 2.05759700

N 8.83863800 -1.95509600 1.26010200

N 7.65533100 -2.50412000 -0.77213500

C 8.65571500 -1.91403200 -0.04659500

C 9.53359500 -1.17009200 -0.93012700

C 10.67929800 -0.41716700 -0.69617100

H 11.07963900 -0.30993700 0.31023600

C 11.28735600 0.17867700 -1.79411500

H 12.18882600 0.77258300 -1.65338200

C 10.76532600 0.02448000 -3.08864600

H 11.27213100 0.50380400 -3.92460600

C 9.62216700 -0.73053700 -3.31980800

H 9.21229600 -0.86190200 -4.31949600

C 9.01395900 -1.32387500 -2.21891700

C 7.83977000 -2.16600000 -2.08709500

N 7.10785600 -2.52240900 -3.12416300

N 5.57014300 -3.89461300 -1.86324900

C 6.07628600 -3.33572500 -3.00746500

C 5.30387000 -3.77659200 -4.15307300

C 5.41094800 -3.50799000 -5.51382700

H 6.19161300 -2.84979900 -5.89067400

C 4.49096700 -4.11405100 -6.35979700

H 4.54343700 -3.93119100 -7.43174700

C 3.49413200 -4.96818200 -5.85973400

H 2.79397900 -5.42882700 -6.55468500

C 3.39148600 -5.23841300 -4.50118900

H 2.62897500 -5.90598900 -4.10417200

C 4.31277800 -4.62855900 -3.65545100

C 4.50264700 -4.67769300 -2.21864800

N 3.71088600 -5.37219000 -1.42312800

N 4.82868200 -4.73141900 0.62092200

C 3.88389500 -5.39715700 -0.11569200

C 3.03329100 -6.17703200 0.76317400

C 1.95758100 -7.02442800 0.51618700

H 1.60770100 -7.19797000 -0.49998400

C 1.36075200 -7.63684400 1.61152200

H 0.51510900 -8.30537800 1.46038500

C 1.82993500 -7.41200600 2.91645500

H 1.33819900 -7.91212100 3.74894700

C 2.90632600 -6.56746200 3.16039400

H 3.28347700 -6.39354400 4.16646100

C 3.49956100 -5.95149600 2.06241200

C 4.62535000 -5.04389900 1.93886000

N 5.31773900 -4.63524600 2.98432500

Co -6.34176700 -3.09200300 0.30274100

N -8.09899500 -2.77562000 -0.42602500

C -8.49918000 -3.05045000 -1.70710100

C -9.86940800 -2.61200700 -1.90049200

C -10.71985100 -2.65625800 -2.99941000

H -10.38653000 -3.08190900 -3.94392000

C -11.99904300 -2.13780000 -2.83807500

H -12.69483900 -2.15289900 -3.67516000

C -12.41631200 -1.59663100 -1.61191200

H -13.42749800 -1.20335900 -1.52229600

C -11.56488200 -1.55732400 -0.51399800

H -11.88008300 -1.14474100 0.44254700

C -10.28447500 -2.07212400 -0.68023500

C -9.15436800 -2.19035600 0.22248000

N -9.20528200 -1.77379900 1.47242900

N -6.93447000 -2.39989000 2.00528900

C -8.17466600 -1.89164100 2.28893200

C -8.21573500 -1.43014400 3.66326300

C -9.22390000 -0.85205100 4.42863200

H -10.21153100 -0.67679600 4.00626100

C -8.91826000 -0.52108900 5.74260500

H -9.68153900 -0.07196500 6.37578800

C -7.64147300 -0.76060600 6.27689700

H -7.44195000 -0.49291400 7.31324900

C -6.63657600 -1.33742600 5.51097800

H -5.64560100 -1.53299000 5.91649300

C -6.94406500 -1.66664200 4.19466500

C -6.16346800 -2.27087400 3.13262100

N -4.89844000 -2.60551800 3.28977300

N -4.62727400 -3.53915300 1.07836500

C -4.20392300 -3.19365800 2.33432300

C -2.81461400 -3.57089100 2.50621700

C -1.93269700 -3.43629700 3.57424300

H -2.26009900 -2.98747800 4.51043700

C -0.63512800 -3.89849100 3.39487900

H 0.08671100 -3.81490700 4.20617200

C -0.23053700 -4.48281800 2.18331200

H 0.79374000 -4.83757200 2.07888000

C -1.11659900 -4.62813900 1.12298200

H -0.81237700 -5.09228900 0.18587000

C -2.41505200 -4.16074200 1.30211500

C -3.57387400 -4.12812700 0.43053000

N -3.54311000 -4.59576800 -0.80415100

N -5.80783000 -3.94784800 -1.34341400

C -4.58962300 -4.52508300 -1.60405500

C -4.57067000 -5.04101700 -2.95960600

C -3.59568700 -5.70505500 -3.69678800

H -2.62723800 -5.93666900 -3.25735300

C -3.91268200 -6.05972400 -5.00212600

H -3.17754100 -6.58378000 -5.61061700

C -5.16851100 -5.76063400 -5.55487300

H -5.38081800 -6.05718900 -6.58060100

C -6.14146900 -5.09946200 -4.81597100

H -7.11984100 -4.86663400 -5.23176300

C -5.82128800 -4.74423100 -3.50993500

C -6.57220000 -4.06069600 -2.47383200

N -7.80610300 -3.63480100 -2.66360300

N -5.46894900 -1.05774600 -0.29708900
